# Perseverance, partnerships and passion: ingredients for successful local government policy to promote healthy and sustainable diets

**DOI:** 10.1186/s12889-023-16656-x

**Published:** 2023-09-11

**Authors:** Liza R. Barbour, Julie L. Woods, Julie K. Brimblecombe

**Affiliations:** 1 Department of Nutrition, Dietetics & Food, Monash University, Level 1, 264 Ferntree Gully Road, Notting Hill, VIC 3168 Australia; 2 Institute for Physical Activity and Nutrition (IPAN), School of Exercise and Nutrition Sciences, Deakin University, Locked Bag 20000, Geelong, VIC 3220 Australia

**Keywords:** Food sustainability systems, Public policy, Local government, Healthy and sustainable diets, Implementation research, Planetary health

## Abstract

**Background:**

Local government authorities are well-placed to invest in evidence-based food policies that promote a population-wide shift to healthy and sustainable diets. This study describes the contextual factors that facilitated or impeded policy-making related to healthy and sustainable diets within a ‘best-performing’ local government in Victoria, Australia.

**Methods:**

Guided by the Consolidated Framework for Implementation Research (CFIR), data from semi-structured interviews with individuals involved in developing the City of Greater Bendigo’s Food System Strategy were analysed using the seven-stage Framework Method.

**Results:**

Semi-structured interviews (*n* = 24) were conducted with City of Greater Bendigo employees (*n* = 15) and key stakeholders working for local organisations (*n* = 6) or at a state or national level (*n* = 3). Interviewees mostly held positions of leadership (*n* = 20) and represented diverse areas of focus from health (*n* = 7), food systems (*n* = 4) and planning and public policy (*n* = 3). Data analysis revealed 12 cross-cutting themes; eight facilitating factors and four impeding factors. Facilitating factors included perseverance, community engagement, supportive state policy, effective leadership, a global platform and networks, partnerships, workforce capacity and passion, and the use of scientific evidence. Impeding factors included access to secure, ongoing financial resources, prohibitive state and federal policy, COVID-related disruptions to community engagement and competing stakeholder interests. Overall, this study suggests that the City of Greater Bendigo’s success in developing an evidence-based local food system policy is built upon (i) a holistic worldview that embraces systems-thinking and credible frameworks, (ii) a sustained commitment and investment throughout the inner-setting over time, and (iii) the ability to establish and nurture meaningful partnerships with community groups, neighbouring local government areas and state-level stakeholders, built upon values of reciprocity and respect.

**Conclusions:**

Despite insufficient resourcing and prohibitive policy at higher levels of government, this ‘best performing’ local government in Victoria, Australia developed an evidence-based food system policy by employing highly skilled and passionate employees, embracing a holistic worldview towards planetary health and harnessing global networks. Local government authorities aspiring to develop integrated food policy should nurture a workforce culture of taking bold evidence-informed policy action, invest in mechanisms to enable long-standing partnerships with community stakeholders and be prepared to endure a ‘slow-burn’ approach.

**Supplementary Information:**

The online version contains supplementary material available at 10.1186/s12889-023-16656-x.

## Background

The health of current and future generations is dependent on wise stewardship of the natural systems on which human civilisation depends [1]. Local governments (LGs), at the interface between civil society and higher levels of government, are well-positioned to lead this stewardship towards planetary health [1–3]. The food system, defined as “the interconnected system of everything and everybody that influences, and is influenced by, the activities involved in bringing food from farm to fork and beyond” [4], p1), is currently contributing to chronic disease, climate change and social inequities and requires radical transformation [5–8] In order to promote human health within planetary boundaries, a whole-of-system approach is required to change the way food is both produced and consumed [9]. While this requires global-level action, leadership must also occur at the local level [3].

Local governments globally are investing in a variety of policy actions to facilitate a population-wide shift towards healthy and sustainable diets [10, 11]. Evidence suggests that having a dedicated food policy is an effective approach for LGs to progress their food system work, however only some are taking this approach [3, 10, 11]. Hawkes and Halliday (2017) define LG food policies as “a concerted action on the part of [city] governments to address food-related challenges”, with documented goals and a description of the various policy actions and key stakeholders required to achieve these [12]^, p9^. Missing from the literature is an understanding of the processes and mechanisms used by LGs to develop these local food policies in the first place, including the contextual factors that facilitate or impede evidence-based policy making. In an Australian study of LGs, Carrad et al. (2022) identified a number of barriers including access to human resources, funding and food system work not being considered an organisational priority [13]. They also revealed that policy-makers prioritised an individual-focused approach to shifting consumption patterns [13]. Such an individualistic approach separates consumer behaviour from the surrounding context and is unlikely to achieve a long-lasting shift in population diets [14]. Policy must adopt a more holistic approach that considers the complex, interconnected nature of the food system [5, 7, 14]. Such integrated food policy can trigger transformation throughout the food system by promoting specific diet-related practices that improve where people source their food, what they eat and how they consume their food [15].

This study draws upon the field of implementation science to explore the way contextual factors either facilitate or impede the policymaking process to support healthy and sustainable diets. Implementation science is the scientific study of methods and strategies used to promote the systematic uptake of evidence-based interventions, with the overall aim to improve population health [16, 17]. While evidence is abundant regarding which policy actions must be invested in [12, 18–24], less is known about the intricacies involved in the policymaking processes, specifically the way an evidence-based food policy is developed to shift population diets towards those that promote human and planetary health. This study therefore aims to describe how contextual factors influence the way policy is prioritised and actioned by a ‘best performing’ local government authority to facilitate the uptake of healthy and sustainable diet-related practices.

## Methods

### Study setting

Australia’s public governance system comprises three levels; federal, state and local, with local government sitting closest to civil society. This study was conducted in the state of Victoria in Australia’s south east, which contains 79 local government areas including the City of Greater Bendigo (CoGB). The CoGB is a regional centre in Victoria’s north, the state’s third largest urban area and has an estimated population of 120,000 people living amongst productive agricultural land.

The CoGB has been ranked by Australian scholars as a ‘best performer’ in healthy, equitable and environmentally sustainable food system policy action [25, 26], largely based on the development of their comprehensive, integrated and evidence-based food system strategy. Greater Bendigo’s Food System Strategy 2020–2030 was established by the CoGB in partnership with over 30 organisations and community groups. In addition, the CoGB was admitted to the UNESCO Creative Cities Network in 2019 in the category of Gastronomy, recognising its work to *‘celebrate and elevate First Nations’ culture, creativity and knowledge’* and *‘prioritise environmental sustainability, sustainable agriculture and food production’* amongst other commitments [27]. The CoGB was also the first Australian local government authority to sign the Glasgow Food and Climate Declaration.

### Study design

To examine the policymaking process through multiple perspectives, an interpretivist lens was applied where findings emerge from the researchers’ interaction with the participants, and “all of the (different) interpretations are considered contextually dependent on the history and culture that influences how each individual interprets and makes meaning of their world” [28]^, p7^. Qualitative methods were chosen to allow an examination of the various key stakeholder perspectives, while allowing for the perspectives and backgrounds of the researchers involved to contribute to the research findings [29, 30]. The lead author (LB) has worked in public health nutrition since 2003 and has pre-existing knowledge of Victoria’s policy landscape. Coinvestigators (JW and JB) each have extensive expertise in food systems research and food and nutrition policy practice.

### Theoretical framework

Data collection and analysis were informed by the Consolidated Framework for Implementation Research (CFIR), which is a conceptual, meta-theoretical framework that provides a comprehensive listing of constructs found to influence implementation [31]. The CFIR is intended to guide a systematic analysis of these constructs or ‘factors’, to identify those that facilitate or impede evidence-based practice. The CFIR has thirty-nine constructs each sitting within one of five major domains: i) intervention characteristics; ii) outer setting; iii) inner setting; iv) characteristics of individuals; and v) process.

### Sampling and recruitment

Purposive sampling was guided initially by two CoGB employees and subsequently through peer-nomination snowball sampling [32]. Individuals, or *key informants *[33], were deemed eligible for an interview if they were involved in the development, implementation or evaluation of Greater Bendigo’s Food System Strategy (2020–2030). Twenty-six eligible individuals were invited via email to participate in an interview, with a follow-up email sent if a reply was not received after two weeks. Interviewees were categorised as either: i) CoGB employees; ii) external stakeholders working within the CoGB jurisdiction; or iii) external stakeholders working at a state or national level. Interviewees were further categorised according to their level of management based on job title whereby *senior management* included elected representatives, managers and directors, *mid-management* included executive officers, senior officers and coordinators and *project officers*.

### Data collection

This study was approved by the Monash University Human Research Ethics Committee (Project ID: 28,221) with permission granted from the CoGB to be involved in the recruitment of participants and to be publicly named in published research and translation activities. Each interviewee provided written consent to participate in a semi-structured interview via Zoom or telephone. An interview guide *(A1: Interview Guide)* was pilot tested prior to use, and amended iteratively throughout the interview process. The line of questioning was informed by the CFIR, designed to prompt interviewees to describe the contextual factors that facilitated policy-making, and to identify enablers and barriers to the prioritisation of the previously published 13 healthy and sustainable diet-related practices [15]. These 13 specific individual practices were identified to trigger broader food system transformation, for example connecting with primary food producers, consuming no more than recommended animal-derived foods and adopting food waste-minimisation strategies *(A2: Healthy and Sustainable Diet-Related Practices)*.

### Data analysis

Interview transcripts were uploaded into NVivo [34] qualitative research software and systematically analysed using Gale et al.’s (2013) seven-stage Framework Method [35, 36]. This method offers credibility by ensuring rigour and transparency in the analysis of qualitative data [35, 36]. This method was chosen as it allows for a holistic, descriptive overview of an entire data set without losing the context of each participants’ views [25, 36]. These steps are detailed in A*3: Step-wise Approach to Data Analysis* however in summary, Step 1 involved post-interview reflection using “face sheets” and verbatim transcription of each interview recording [37, 38]. Face sheets were used to summarise key data for each interviewee including name, the length of interview, any special circumstances or contextual issues that may have impacted the interviewer or interviewee on the day [37]. Following each interview, a summary was written of key topics described in the interview and any items discussed that required follow-up [37]. Step 2 enabled familiarisation with the interview by updating the summaries of key findings within the face sheets for each interview and returning the transcripts to interviewees for member-checking [39] purposes. Step 3 involved independent, double-coding of two transcripts by LB and either JB and JW, using the CFIR analytical framework. Step 4 enabled refinement of the analytical framework and its testing via independent coding by LB and JB of one transcript. The five domains of the CFIR were maintained as they remained relevant to the research questions and interview data, however the constructs within each domain were adapted accordingly. Step 5 applied this framework in NVivo whereby LB conducted line-by-line coding of all transcripts, iteratively updating the framework to accurately define and name each construct within the five domains *(A4: Coding Framework)*. Step 6 charted this data into a matrix in Microsoft Excel, with illustrative, verbatim quotes exported from NVivo into the matrix. Step 7 interpreted this data using a two-fold thematic analysis approach [40], firstly to identify sub-themes within each of the constructs and secondly to identify cross-cutting themes that spanned multiple constructs within one or more domains. These cross-cutting themes were discussed by all authors and visualised to present them as facilitating factors, impeding factors and in some cases, both. Interviewees were invited to comment on the results of this study at two time points. Firstly, at a stakeholder workshop in May 2022 where preliminary results were presented orally, and secondly, two weeks prior to submission of the manuscript a draft was sent to all interviewees.

## Results

Twenty-four semi-structured interviews were conducted between May and September in 2021 (response rate 92%). Interviews were conducted by videoconference (Zoom) (*n* = 22) or telephone (*n* = 2) and ran for an average duration of 52 min. Of the 24 participants interviewed, 15 were CoGB employees, 6 were external stakeholders working within CoGB’s jurisdiction and 3 were external stakeholders working at the state or national level *(*Table 1*)*. While the 15 interviewees employed by the CoGB reflected on their first-hand experience at the heart of the policymaking processes, the 9 interviewees that weren’t employed by the CoGB provided a more distanced perspective as majority worked across multiple municipalities. The majority of participants were employed at a mid-management (*n* = 11) or senior management level (*n* = 9), including elected representatives, managers and directors. Participants were employed in roles with diverse areas of focus, with the most common being health (*n* = 7) and food systems (*n* = 4).

**Table 1:** Study population

*Category*	*Descriptor*	*Total* *(n* = *24)*
*Employee and geographic remit of role*	*CoGB employees* *External Stakeholders – working in CoGB’s area* *External Stakeholders – working at the state or national level*	*15* *6* *3*
*Level of current role*	*Senior Management* *Mid-Management* *Project Officer*	*9* *11* *4*
*Area of Focus*	*Population Health* *Food Systems* *Planning and public policy* *Water Strategy* *Community House* *Food Rescue & Relief* *Circular Economy* *Agribusiness* *Nutrition* *Education* *Regional Sustainable Development* *Food Safety*	*7* *4* *3* *2* *1* *1* *1* *1* *1* *1* *1* *1*

**Fig. 1:**
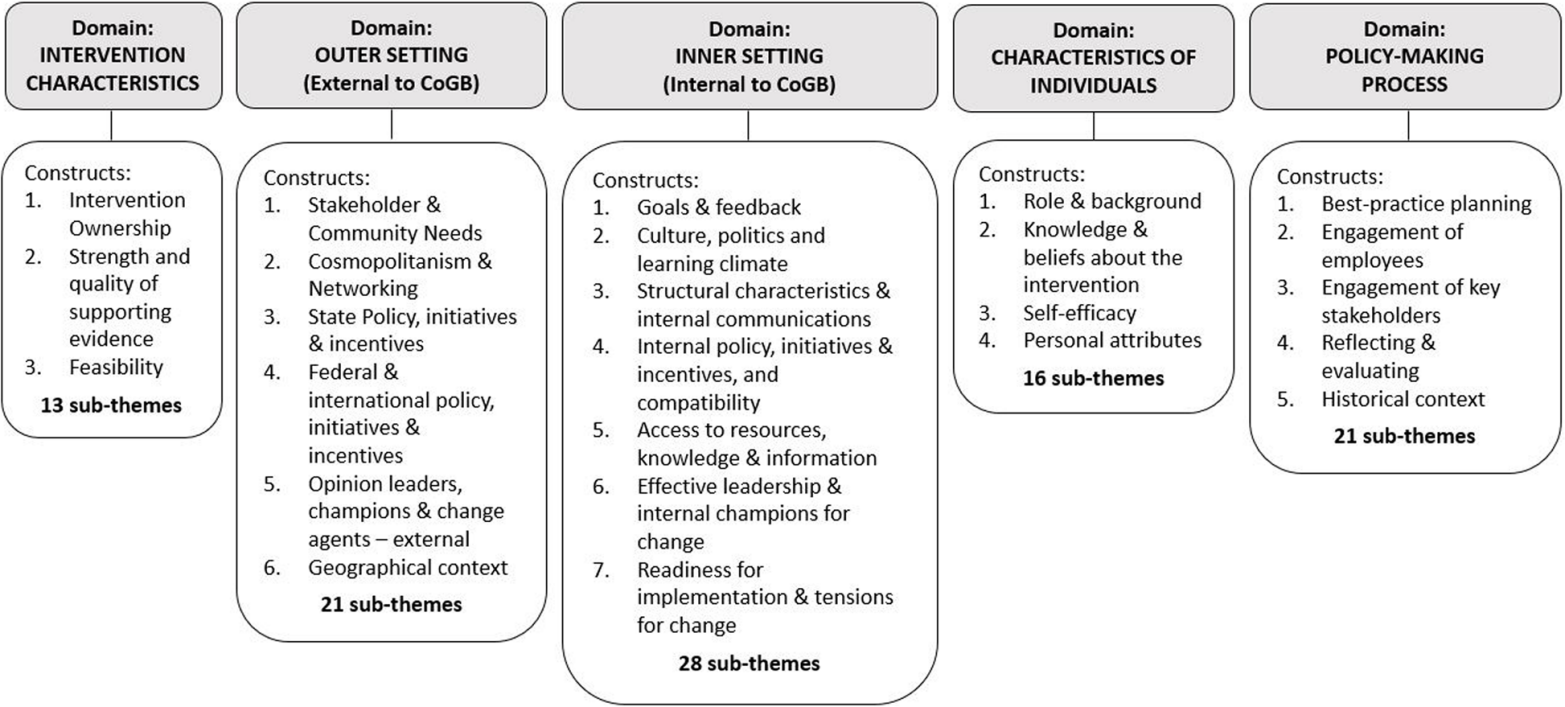
Coding framework, informed by the Consolidated Framework for Implementation Research

The final analytical framework comprised 25 constructs or ‘factors’ considered to be relevant to the way CoGB’s policy activities were prioritised and actioned *(*Fig. 1*: Coding framework)*. Thematic analysis of data coded to each construct revealed 99 sub-themes across the five domains (*A5: Sub-themes and Examples of Illustrative Quotes for each Construct, organised by Domain)*.

**Fig. 2:**
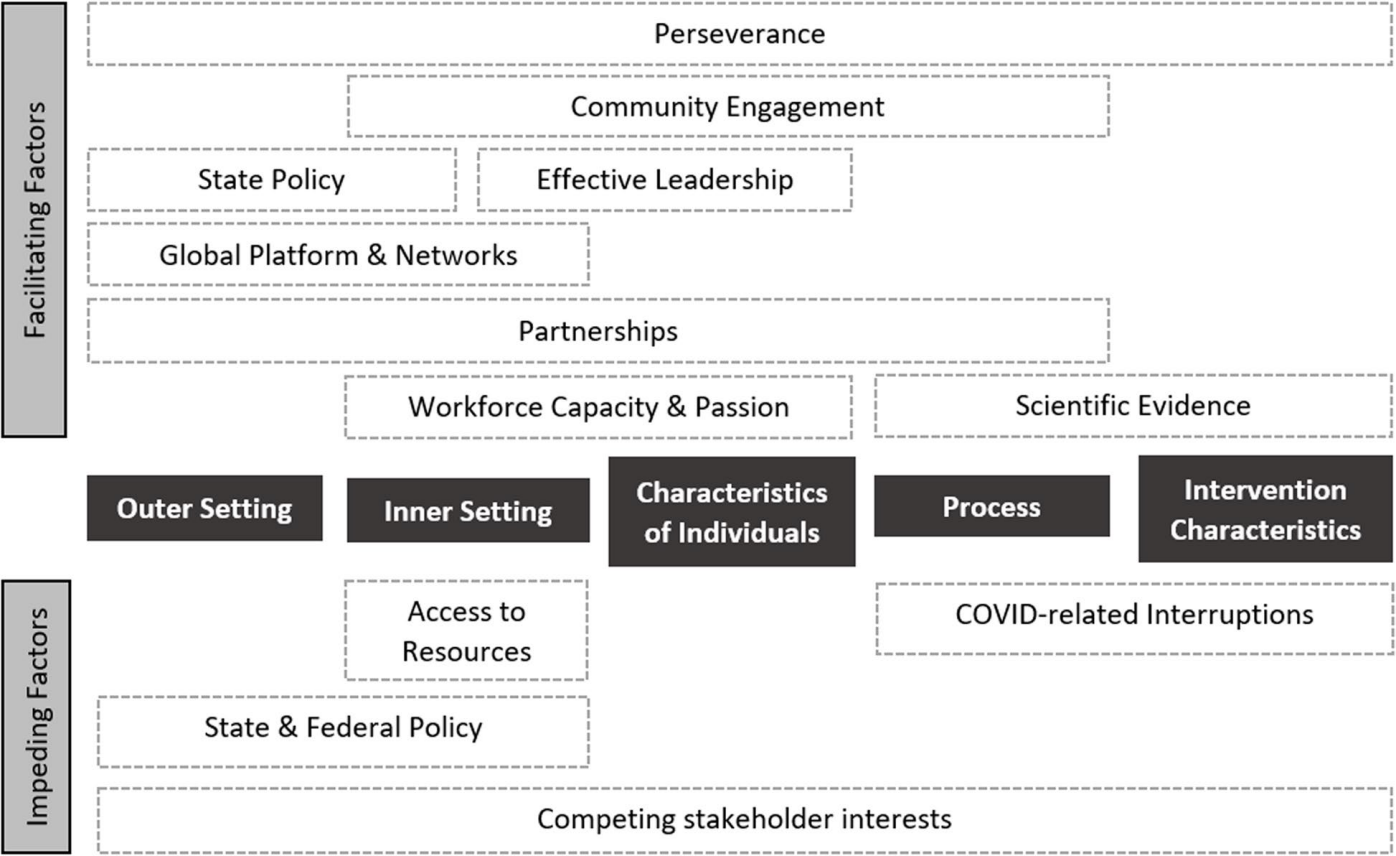
Cross-cutting themes—facilitating and impeding contextual factors across the five domains of the Consolidated Framework for Implementation Research

Within these sub-themes, 12 cross-cutting themes were identified, of which eight were considered to facilitate evidence-based policy action, while four were deemed to impede such action *(*Fig. 2*: Cross-cutting themes—facilitating and impeding contextual factors)*. As illustrated, some cross-cutting themes are relevant to a single domain while others transcend multiple domains. State policy was described as both a facilitating and impeding factor, cutting across both Outer and Inner Setting domains.

### Facilitating factors

#### Perseverance

The theme of perseverance spanned all five domains and captured the relentless passion and drive for policy action. Perseverance in creating healthy, equitable and sustainable food systems was described in relation to the people driving action as well as the enduring policies within CoGB and at the state level. In considering the CFIR domains of outer setting and process, the outer and historical state-level food system initiatives such as Healthy Together Victoria (2011–2016), VicHealth’s Food for All (2005 – 2010) and Water in Sport project (2018–2020) were said to enable the food system activity that has persisted in CoGB. This policy context enhanced inner workforce systems-thinking capacity and evidence-based public health practice, enabled the employment and retention of highly skilled employees, and established a solid evidence base to inform future work. For example, Food for All established a clear picture of food insecurity in the area that in turn contributed towards evidence-informed policy-making.

*“Probably 10 years ago, someone might say that food and healthy eating wasn't really a big thing. But thanks to a few (passionate leaders), they've been able to change the conversation. So, it's probably been a slow burn issue, but they've just kept at it and kept working at it.” COGB Employee, Mid-Management*

In this process of driving change over the years, a number of pivotal events and pieces of work championed by CoGB were mentioned by interviewees as contributing to the current food sustainability agenda, including their UNESCO City of Gastronomy application and their contributions to the People’s Food Plan (Australia). The perseverance of passionate actors within CoGB to make these events happen, and to embrace emerging best-practice evidence and partner with academic institutions, helped increase their credibility with external organisations and the community.

#### Community engagement

Engagement with local residents, primary producers and community organisations was described as a facilitating factor for policy action, particularly in the development phase of the Food System Strategy. CoGB employees talked about the legal and moral imperative to spend public funds on what their community wants, and described this as a key motivator to establish mechanisms, as part of the food system strategy, to identify and respond to community need. Such mechanisms included a food system strategy reference group comprised of external stakeholders, a pre-existing network of local primary food producers (Farming and Agribusiness Advisory Committee) and a stakeholder workshop. Other benefits were seen to flow from investing in these community engagement activities including minimising potential duplication of activity, smoother execution of policy action plans and an ability to understand and address tensions and pushback by communicating directly with the community. Several external stakeholders acknowledged that while the Food Systems Strategy is led by CoGB it is owned and implemented by many. Several interviewees attributed this to the diligence of key champions working within the CoGB, who built upon historical partnerships with relevant community members and stakeholder groups to implement a comprehensive and ‘best-practice’ consultation process for developing the Food System Strategy. Bendigo’s regional location and *‘small-town community’* size was also thought to be more conducive to meaningful community engagement. CoGB was seen by several external stakeholders as having the ability to engage community at the grassroots level, meeting them *‘where they’re at’* then facilitating the transition towards systemic change.

#### Effective leadership

This cross-cutting theme of effective leadership spans both the *inner setting* and *characteristics of individuals* domains. Within the CoGB, various leaders have instilled a culture of taking bold and evidence-informed action, adopted new ideas, shared their implementation experiences with others, and exercised agility regarding new areas of focus by applying an environmental sustainability lens over existing health policy activities. For example, CoGB embraced the arts to achieve food systems change through UNESCO’s Creative Cities network.

*“Absolutely, good quality workforce that’s surrounded by opportunity. The leadership in the town is willing to try things and to be innovative.” External Stakeholder (Local), Senior Management*

Interviewees described their leaders and managers within CoGB as committed, competent and accountable to the food sustainability agenda, using words such as “*passionate”*, “*inspiring”, “trailblazer”* and *‘a thorn in the side’* (in relation to persistent advocacy efforts). There was a recognised history of CoGB’s employment of highly skilled employees with extensive and diverse leadership experience having worked in academia, health services and federal and state-level advocacy roles. For example, the Director of Health and Wellbeing who was soon to manage the Environment team was a Dietitian and the Mayor has a doctorate of public health and extensive experience in urban agriculture, community food hubs and the food relief sector. Another leader used their expertise to lead CoGB to the UNESCO City of Gastronomy designation that further built leadership capacity through connecting the organisation with global leaders.

#### Global platform and networks

Spanning both the *outer setting* and *inner setting* domains, the UNESCO City of Gastronomy work played an important role in amplifying and strengthening CoGB’s food system strategy work. The new connections with global leaders, prompted innovative thinking on local food system challenges.

*“We know that we've actually attracted new business … a couple of restaurants have moved here since. And there's a whole heap of other things that have… been made more visual…we've now got a platform to actually communicate it. We were already on the path … we had the food system strategy I think what the Gastronomy stuff does is really give us a higher-level order, sort of audience, but also (an) authorising environment to really push things.” CoGB Employee, Senior Management*

The application process itself documented CoGBs existing and aspirational targets, strengthened local partnerships (eg. with local Dja Dja Wurrung community members) and identified the health, tourism and economic outcomes of their food system work. CoGB employees described their involvement in UNESCO’s Creative Cities network as reinforcing the existing workplace culture of reciprocity, whereby CoGB employees were keen to contribute and support others in the network and their global citizenship.

#### Partnerships

Partnerships as a theme, builds upon the previous themes of perseverance, community engagement and the global platform. Numerous examples were given of the historical investment of CoGB in long-standing partnerships with community groups, local organisations, neighbouring LGAs and state-level stakeholders, each considered critical to achieving an integrated approach to food policy.

*“What we've gradually done by building partnerships, and understanding and credibility, we're gradually taking them on a journey towards food. But you've got to start where people are at - you have to take people on this sort of thinking journey.” COGB Employee, Senior Management*

In relation to the City of Gastronomy work, interviewees mentioned new partnerships with international local government authorities, specifically Östersund in Sweden, San Antonio and Tucson in the US, and several Italian Cities of Gastronomy, and also several Australian local government authorities. For example, CoGB partnered with the City of Launceston to support their successful application to join CoGB as a City of Gastronomy in 2022. Benefits from investing in these partnerships included the ability to collect and communicate reputable data by partnering with academic institutions, increased workforce capacity, success with competitive funding, broadened recognition of their work, and successful recruitment and retention of a skilled workforce.

#### Workforce capacity and passion

Capacity and passion of the CoGB workforce were described as key enabling factors to progressing the food sustainability agenda and policy action. These spanned both the *inner setting* and *characteristics of individuals* domains. CoGB employees were described as well-versed in the complexity of food policy for system transformation, humble enough to seek expert involvement where required, and highly experienced in health, nutrition and dietetics, food safety regulation, circular economy, sustainable development, agribusiness, water management, leadership roles (e.g. Chief Executive Officers), academia, legislation and community development.

*“I think it comes in a big part down to governance. Certainly, we've had some real trailblazers, some of the directors and managers that we've had on board have certainly been real leaders in that field… certainly their backgrounds have influenced the way that they view the world and the paradigm for which they see it.” COGB Employee, Mid-Management*

The size and geographical location of Bendigo was seen as an enabler to attracting a skilled workforce. In addition to skill and experience, the passion amongst employees was considered an important enabler, particularly in enabling the perseverance over time as described previously. Interviewees described their personal motivations to promote health and environmental sustainability within their work responsibilities. On a self-ranked scale of one to five (whereby one is no interest in adopting healthy and sustainable diet-related practices at home and five is to live and breathe these practices in all interactions with the food system), all interviewees ranked themselves as at least three of five. Their motivations were to support local producers, contribute to the local economy, increase local employment, enjoy being active participants in the local farmers’ market and making social connections with other like-minded individuals, with several interviewees growing up on food-producing farms.

#### Scientific evidence

A commitment of the CoGB to evidence-based practice both in the way the food systems strategy was developed (*process* domain) and its policy activities (*intervention characteristics* domain) was described by interviewees. A number of frameworks were mentioned that informed practice, including One Planet Living principles [41], systems change framework [42, 43] and the Collective Impact model [44]. CoGB employees described their role in monitoring and evaluation whereby the food system strategy aims to establish a system that favours healthy, sustainable and equitable food then CoGB steps aside and focuses on ensuring the system is continually evaluated and remains on track. Interviewees referred to CoGB’s investment in local data collection via their Active Living Census, first conducted during the Healthy Together Victoria implementation phase (2011–2016). The value of this local data was mentioned by several external stakeholders as informing their work prioritisation and internal policy agendas.

*"In Healthy Together (Victoria), they did the first Active Living census. So, we also had data, which was fabulous. And so, it's very hard to argue with the amount of data that we had, and … the whole census was done by the Social Research Centre. It was reputable data. And we've leveraged it within an inch of its life, really, and then used it to go and do more consultation and so forth." COGB Employee, Senior Management*

#### State policy

State-level policy was said to both facilitate and impede the CoGB to progress their food sustainability agenda. While state-level policy sits within the outer setting domain, its influence on day-to-day practice falls within the inner setting. Several state government interventions were said to provide a supportive influence such as Hospital Procurement Victoria, VicHealth’s Water in Sport, Healthy Together Victoria, Healthy Choices Guidelines, Victorian Population Health Survey collection, Nutrition Australia’s Healthy Eating Advisory Service, Victoria’s Achievement Program, INFANT and Healthy Food Connect. Many CoGB employees mentioned Victoria’s Health and Wellbeing Act and the Local Government Act as being a critical driver for their food sustainability work. This Act mandates all Victorian LGAs to report on their policy activities that consider the inter-related impacts of health and climate change to the Victorian government in four-yearly cycles. This reporting responsibility was described as one of the reasons why all interviewees considered local governments to be responsibility for addressing both health and environmental sustainability outcomes simultaneously.

### Impeding factors

#### Inadequate resources to translate aspirational policy actions into reality

Inadequate resources were described as a barrier to translating aspirational policy ideas into reality. CoGB was considered better resourced than other LGAs that have a smaller rate-payer base and leadership within the CoGB was described as a key enabler to securing funding for dedicated food system officer positions for example. However, interviewees in senior management roles described frustrations at the lack of dedicated funding from higher levels of government. They attributed this to a lack of state and federal commitment to food system transformation more broadly, that undermined the feasibility of local governments to prioritise and effectively execute food systems activities. There was a fear or hesitation amongst interviewees that without adequate resourcing and support from state government, their food system strategy would remain *‘a great ambition’*. Without such investment, CoGB interviewees also considered themselves less well equipped to implement the monitoring and evaluation work that they described as being critical to the effective execution of the strategy.

#### State and federal policy

State policy was considered both a facilitating factor, as described earlier, and an impeding factor. CoGB employees commented that where action and alignment between international, federal, state and local policy action was missing, local governments fill the gap or advocate for higher order change to address the gap. This however was described as adding unnecessary financial and workforce pressure.

*We've identified a problem, and without, to be honest, without the support of any federal real federal statement we've just gone and said we need to do something about it, because you're not.” COGB Employee*,* Senior Management*

CoGB employees discussed the impact of election cycles at state and federal levels in meeting community needs and progressing their stated policies. For example, the cessation of Healthy Together Victoria funding following a change in state government halted significant state-wide progress amongst LGAs and promptly eliminated a funded workforce. In addition to the challenges of this dynamic policy landscape, some state legislation items were described as non-progressive and prohibitive in achieving CoGB’s sustainability outcomes. For example, the Class 4 Simple Sausage Sizzle regulation that only allows sausages, bread, sauce and onions, prohibited local government from allowing vegetarian and plant-based options to be offered at fundraising events on public land.

#### COVID-related disruptions

The COVID-19 pandemic was considered an impeding factor across both *process* and *intervention characteristics* domains. Interviewees described stay-at-home orders and social distancing requirements that impacted CoGB’s ability to engage the community, for example attendance dwindled at the Farming and Agribusiness Advisory Committee meetings. The implications of missing this face-to-face engagement with local residents were described as jeopardising CoGBs commitment to reflect community need and community buy-in to both prioritise and action policy. CoGB employees expressed gratitude for the efforts made to establish solid relationships, credibility and respect amongst the community prior to the pandemic. The pandemic also altered the trajectory of their food system strategy work, since employees were required to shift their focus to alleviate acute food insecurity amongst residents affected by the health and economic impacts of COVID-19, rather than strengthen food system sustainability and resilience more broadly.

#### Competing stakeholder interests

A number of tensions in managing stakeholder interests were described, spanning all five domains. CoGB employees described their important brokerage role in managing expectations between state-level stakeholders (*outer setting*) from both government and non-government sectors, where they don’t always align with local community need. CoGB employees also described some tensions or ‘*tussles’* that exist internally (*inner setting*) whereby some employees (*characteristics of individuals*) believe local government should focus on core business rather than progressing the food sustainability agenda.

*“Yeah, there's always detractors who think it's (City of Gastronomy work) just a sort of vanity project, or just wasting time… people saying just focus on roads, rates, and rubbish. And all of this international networking and creative industries stuff is a nice to have but isn't really core business.” CoGB Employee, Project Officer*

In terms of policy development (*process*), interviewees described walking a tightrope between being ‘liked’ by the community and implementing evidence-based, bold and *“controversial”* policy action. CoGB employees described a legal and moral requirement to listen and respond to community need, including the needs of locally-based industrialised producers of chicken and pork, that had proved problematic when trying to align with bold, food sustainability policy actions. Interviewees described this as ‘a bit jarring’ in trying to support a population dietary shift towards less meat and more plants when two of the biggest employers in the region were producing meat using intensive farming practices. CoGB employees also described a resistance to the *‘nanny state message’*, with several interviewees being very reluctant to have residents feel like local government are telling them what to eat.

## Discussion

This research identified a number of contextual factors that influenced the way policy was prioritised and actioned by a ‘best performing’ local government authority (the City of Greater Bendigo in Australia) to promote the uptake of healthy and sustainable diet-related practices. Overall, this study reveals that the City of Greater Bendigo’s success in developing an evidence-based, local food system policy is built upon (i) a holistic worldview amongst policymakers that embraces systems-thinking and credible frameworks, (ii) a sustained commitment and investment towards food sustainability over time, and (iii) policymakers’ ability to establish and nurture meaningful partnerships, built upon values of reciprocity and respect.

### A holistic worldview that embraces systems-thinking and credible frameworks

A key finding was the holistic worldview that had been adopted and nurtured at all organisational levels resulting in a whole-of-system approach to the policy-making process. This holistic worldview relies on systems’ thinking and an intention to improve the overall health of the whole system, requiring involvement from everyone, everywhere to ultimately shift daily interactions and relationships between humans and nature [14, 42, 43]. The CoGB successfully embedded this worldview within their workplace culture over time, and sustained this through the ongoing recruitment of committed and passionate staff and leaders who share a belief in the same approach. The CoGB’s current food sustainability policy reflects a mature, systems-thinking approach that was intrinsic to the holistic worldview. Interviewees described the way CoGB has embedded this systems perspective in their workplace culture, that continues to guide their day-to-day practice both internally and in their work with external partners.

The policy hierarchy surrounding this ‘best performing’ local government supported this holistic approach to their food policy. Having a supportive authorising environment is considered important to implementation success throughout the policy-making process [45]. The Victorian State Government, sitting at one level above the CoGB, has historically been more innovative in food and nutrition policy than other states and territories in Australia [46]. This is evidenced by their historic establishment of VicHealth in 1987, the world’s first health promotion body to be funded by a tax on tobacco [47], and their investment in Healthy Together Victoria (2011–2016), that facilitated a state-wide systems-thinking approach to food policy at the LG level [48]. However, despite this progress Carrad et al. (2022) identified that currently only 11 of the 79 LGs in Victoria have a dedicated food system policy [26]. Our findings suggest that the CoGB may be unique in that they have developed their dedicated food system policy as well as adopted a ‘food in all’ approach, to integrate food sustainability into multiple policies [24]. For example, their Circular Economy and Zero Waste Policy aims to achieve net zero carbon emissions and zero Waste output by 2036. This reflects their commitment to embracing a systems-thinking approach, likely a legacy of the Healthy Together Victoria intervention as well as the organisation’s commitment to evidence-based practice. The CoGB’s systems-thinking approach to food policy may contribute to improvements in various aspects of their internal processes (e.g. more holistic, ambitious waste management policies) and improved outcomes for the community (e.g. health, sustainability and equity). While beyond the scope of this current study, this warrants further investigation.

Credible frameworks and concepts were described by interviewees as being embedded into day-to-day practice. Interviewees referenced a number of well-known theories that recognise the connection between local food policy and the global food system. For example, the One Planet Living sustainability framework that considers social, environmental and economic aspects to enable a world where everyone, everywhere can live healthy lives within planetary boundaries [41]. The CoGB has embedded the ten One Planet Living principles throughout their internal policies, including Greater Bendigo’s Food System Strategy [49]^, p9^, and is working with partners in their region to achieve recognition as a One Planet Living City and Region [3]. CoGB’s commitment to global citizenship, that allows individuals and organisations to embrace their social responsibility to act for the benefit of all societies, was further demonstrated through their commitments as a UNESCO City of Gastronomy and signatory city to the Glasgow Food and Climate Declaration. Our findings demonstrate the ability of these global platforms to reinforce the use of evidence-based frameworks to strengthen local responses to food systems challenges, as previously reported by other scholars [10, 50–52].

### A sustained commitment and investment throughout the inner-setting over time

This study also revealed that the sustained investment in food sustainability over at least two decades was critical to success. Our findings illustrate how this has been enabled through the state-government funded and led initiatives of Food for All (2005 – 2010) and Healthy Together Victoria (2011–2016), both of which have left significant legacies in the CoGB such as the now embedded Active Living Census data collection and retention of key personnel who were employed to initially deliver these initiatives [48, 53]. Through these experiences, CoGB has built a rich, supportive, learning environment over a long period of time and has demonstrated agility in responding to a dynamic policy landscape. Carrad et al. (2022), in a study of employees from six LGs in Victoria and New South Wales (Australia) to understand barriers and enablers to their food system policies and initiatives, also found a supportive state government policy environment to be an enabler for policy-makers in Victoria. Interviewees told us that this translated into internal leadership, a cohesive prioritisation of food sustainability within the inner setting, partnerships with other LGs and local non-government organisations. Limited access to funding from higher levels of government however could undo such progress and was identified as a significant barrier.

The dogged determination and perseverance over time of the policy actors within our study played a critical role in progressing CoGB’s food sustainability agenda, despite the evolving policy and funding landscape. Bullock et al. (2021) describe policy being nested within a context of “ideas (values, evidence, etc.), interests (interest groups, civil society, etc.), institutions (existing rules and institutional structures), and external factors (natural disaster, change in economic conditions)” [45]^, p10^ and acknowledge that these contextual factors change over time, influencing the policy-making process. They describe this constantly evolving context as requiring an agility amongst policy-makers throughout the development and implementation phases. Our results suggest that the CoGB has created a culture where this dynamic process has been embraced with enthusiasm. For example, they have been early adopters in applying the sustainability lens over their existing health and wellbeing policy work [13, 26]. This is in part due to the values and personal interests of individuals employed by the CoGB, which is unsurprising as policy actors themselves are known to play a critical role in responding to this dynamic process [45, 54–57]. While individuals themselves have championed specific activities to progress CoGBs food sustainability agenda, our results suggest that it is the workplace culture that nurtures and celebrates these passionate individuals that has sustained their commitment over time.

### Establishing and nurturing meaningful partnerships, built upon values of reciprocity and respect

Developing integrated food policy cannot be achieved in isolation, and requires policy-makers to work alongside stakeholders within the food system, civil society and partners from a diverse range of sectors. Interviewees described a number of mechanisms used to achieve this in the CoGB including their collective impact approach, where the food systems work is owned by many [44, 58]. They described their effective partnerships with a number of stakeholders, including a local, independent supermarket to improve the healthiness of this food environment. This partnership was part of a randomised control trial [59], further demonstrating CoGB’s commitment to collaborating with researchers to enhance evidence-based practice. Participants also described partnerships with local Aboriginal and Torres Strait Islander peoples, specifically in relation to their successful application for the UNESCO City of Gastronomy designation. It is well-understood that policy-makers must work alongside Indigenous peoples in navigating the path towards planetary health and demonstrate a deep respect for Indigenous peoples as custodians and expert stewards of the land, waterways and our finely balanced ecosystem [1, 5, 60], that was reflected in the way interviewees spoke about their partnership work with members of the local Dja Dja Wurrung and Taungurung communities. It was evident that CoGB fosters a workplace culture where values of reciprocity and respect underpin their work with key stakeholders, with many examples provided of LG employees contributing to networks and generously sharing their learnings with others. An important facilitator for these partnerships was the CoGB’s commitment to networks both locally and on an international platform. These networks facilitated CoGB employees to share their learnings, learn from others and be recognised for their food sustainability policy by a number of key audiences. Their role as ‘knowledge-brokers’ and facilitators was a common thread amongst LG employees interviewed. Although access to government funding was identified as an impeding factor, it may be that this encourages LGs to work in partnership as there was a common understanding that CoGB was dependent on partner organisations to execute their policy action plans.

### Strengths and limitations

A high level of engagement amongst interviewees throughout this research enabled many opportunities to present the preliminary findings and seek input into data interpretation [39]. The rigour involved in applying Gale’s framework of data analysis was another strength, and although the majority of data analysis was conducted by one researcher (LB), the double-analysis involved at several steps of the process allowed for regular checking of researcher subjectivity and bias. The level of detail involved in the Consolidated Framework for Implementation Research meant that developing and refining the initial codebook was resource-intensive. However, the agility and intention of the framework enabled us to tailor the constructs within each domain to be contextually relevant and ultimately have confidence that the influencing factors were analysed in a comprehensive manner. A potential limitation of the study was that interviewees may have presented favourable responses about the policy-making process, knowing that the City of Greater Bendigo would be named in research materials. To minimise this, the interviewer (LB) drew upon existing knowledge from both practical experience and academic evidence [25, 61] to prompt participants to consider both facilitating and impeding factors.

## Conclusions

This study examined the policy-making process within a ‘best performing’ local government authority to reveal factors that facilitated and impeded the implementation of their local food system strategy. Our study offers examples of passionate individuals who have championed the food sustainability agenda over time, culminating in the successful development of their integrated food policy and UNESCO City of Gastronomy designation. Key to this success, was the advocacy work of senior leadership to secure funding for a dedicated Food System Officer position, as well as the skills and attributes of individuals ultimately appointed to these roles. Despite scarce resourcing and commitment from some higher levels of government, the City of Greater Bendigo in Australia embraced holistic, evidence-based ways of working and harnessing local and global networks to inform their work. The insights gained from factors described by study participants that impeded their progress can inform future policy-making, which calls for greater alignment between local, state and federal government policy priorities and with this, more certainty in ongoing funding to resource such food system transformations. Local government authorities aspiring to develop dedicated food system strategies should nurture a workforce culture of taking bold evidence-informed policy action, invest in mechanisms to enable long-standing partnerships with community stakeholders and be prepared to endure a ‘slow-burn’ approach.

### Supplementary Information

**Additional file 1:** **Additional material A1.** Interview guide. **Additional material A2.** Healthy and sustainable diet-related practices. **Additional material A3**. Step-wise approach to data analysis. **Additional Material A4** Coding framework. **Additional material A5.** Sub-themes and examples of illustrative quotes for each construct, organised by domain.

## Data Availability

The dataset supporting the conclusions of this article is included within the article and these five additional materials: • A1: Interview Guide • A2: Healthy and Sustainable Diet-Related Practices • A3: Step-wise Approach to Data Analysis • A4: Coding Framework • A5: Sub-themes and Examples of Illustrative Quotes for each Construct, organised by Domain

## Abbreviations

CoGB: City of Greater Bendigo
LG: Local Government
