# Supplementary material for: Perseverance, partnerships and passion: ingredients for successful local government policy to promote healthy and sustainable diets
Source: BMC Public Health. 2023 Sep 11;23:1762. doi: 10.1186/s12889-023-16656-x (PMC10494407; doi:10.1186/s12889-023-16656-x)
Supplement: Supplementary file 1 — Additional file 1: Additional material A1. Interview guide. Additional material A2. Healthy and sustainable diet-related practices. Additional material A3. Step-wise approach to data analysis. Additional Material A4 Coding framework. Additional material A5. Sub-themes and examples of illustrative quotes for each construct, organised by domain. [file 12889_2023_16656_MOESM1_ESM.docx]

**Additional Materials**

Table of Contents

[Additional Material A1: Interview Guide 2](#_Toc120647088)

[Additional Material A2: Healthy and Sustainable Diet-Related Practices 5](#_Toc120647089)

[Additional Material A3: Step-wise Approach to Data Analysis 6](#_Toc120647090)

[Additional Material A4: Coding Framework 7](#_Toc120647091)

[Additional Material A5: Sub-themes and Examples of Illustrative Quotes for each Construct, organised by Domain 8](#_Toc120647092)

## Additional Material A1: Interview Guide

This is the line of questioning used when interviewing CoGB employees. An adapted version of this was used when interviewing external stakeholders to ensure the questions were relevant to their positioning.

| *Questions* | *Prompts* |
| --- | --- |
| **INTRODUCTION**  *Before starting the interview, do you have any questions?* | |
| To get started, could you tell me a bit about yourself, starting with your background and current role with CoGB? | How long have you worked with CoGB? |
| **ROLE OF LOCAL GOVERNMENT POLICY**  *As you know, there are different ways to define our food system. During this interview, I’m referring to the interconnected system of activities which bring food from farm to fork and beyond, including everything and everybody that influence, and is influenced by these activities (Parsons et al (2019) What is the food system? Centre for Food Policy).* | |
| Experts have advised that we must transform this food system, including a shift in population diets. Can you describe in your own words why experts say this transformation is urgently required? |  |
| My research is focusing on the way this food system shapes our dietary behaviour. What role do you see local governments playing in shifting population diets as part of this broader transformation? |  |
| Do you believe that local governments should consider both health and environmental sustainability outcomes? | Do you believe these outcomes to be connected?  Why do you believe this? |
| CoGB is trailblazing in this area of food policy compared to other Victorian local governments. Why do you believe this is the case? | Any particular stakeholders? Historical policy action? |

| **DIET-RELATED PRACTICES**  *I recently reviewed reports from the United Nations to identify dietary behaviour that can be promoted through public policy to improve health and sustainability outcomes. I’m going to share my screen now to show you the results. <Show Infographic and list the 13 practices> As you can see, these 13 dietary behaviours describe the way we can source our foods, what we can eat and how we can consume our food to have the best outcomes for human and planetary health.* | |
| --- | --- |
| It’s difficult to address all of these practices equally so I’m interested to understand why some of these practices are prioritised over others. What factors are in place that have influenced this prioritisation? | Consider factors internal or external to CoGB and those at local, state, national and international levels. |
| What role has CoGB played in developing and implementing these so far? | Is it one of leadership, partnership or support of community organisations and residents? |
| The next few questions will explore one of these behaviours which has been prioritised by Bendigo. You can choose one of these that you’re most comfortable talking about. Are you aware of any key reasons why this has been prioritised? | What is in place to support this response? e.g. particularly convincing evidence, demand from residents |
| Can you think of any key stakeholders (internal or external to CoGB) who have been critical to this policy response? | Who and how? |
| Can you think of any other initiatives, incentives or mandates which have prompted this policy response? | How did these influence the way certain dietary behaviours have been prioritised? |
| Turning now to some of the barriers. Can you think of one of these practices that has not been prioritised by Bendigo? Can you think of any reasons why this particular behaviour has not been prioritised? |  |
| Can you think of any key stakeholders (internal or external to CoGB) who would need to be on board for this to be prioritised? | What would their role be? |
| Can you think of any factors that would need to be in place for this behaviour to be prioritised? | For example, any initiatives, incentives or mandates? Can be internal or external to CoGB and may exist at local, state, national and international levels. |
| **INDIVIDUAL CHARACTERISTICS**  *As you know, individuals play an important role in the way policy is created and implemented.* | |
| Do you have a personal interest in food, health and/or environmental sustainability? | On a scale of zero to five, to what extent does the sustainability of our food system influence your day-to-day life, where zero is very little and five is a lot? |
| How do you believe this has influenced your work with CoGB and the subsequent policy response? | Could you please explain in more detail? |
| **CLOSE**  *To finish up, let's imagine that you work for a utopian local government, where they have transformed their local food system so that residents adopt all 13 of these desired dietary behaviours.* | |
| Can you describe one or two factors that are in place to achieve such progressive policy action? | Could you please explain in more detail? |
| That’s the last of the official questions. Before we finish up, do you have anything else you’d like to add? |  |
| Just finally, can you think of anyone who has been involved in the food policies we’ve been talking about today who may be interested this study? They can be internal or external to CoGB, they just have to have been involved in developing, implementing or evaluating the relevant food policy. Anyone you suggest will be invited to participate in an interview, however is under no obligation to accept my invitation. |  |

## Additional Material A2: Healthy and Sustainable Diet-Related Practices

| **Where to source food?**   - Select food grown using sustainable food production practices, valuing and respecting Indigenous knowledges - Strengthen local food systems by connecting with primary producers - Eat seasonally, incorporating native and wild-harvested foods - Eat locally available foods |
| --- |
| **What to eat?**   - Avoid over-consumption beyond caloric requirement - Consume no more than recommended animal-derived foods - Limit intake of ultra-processed, nutrient-poor and over-packaged food - Increase intake of plant-based foods - Eat a wide variety of foods to promote biodiversity |
| **How to eat?**   - Adopt food waste-minimisation strategies - Preference home-made meals and share with others - Consume safe tap water as preferred drink - Breastfeed infants where possible |

## Additional Material A3: Step-wise Approach to Data Analysis

| *Step 1: Transcription* | Face sheets were completed immediately after each interview and added to during the transcription process, to document the researcher’s (LB) personal reflections on the interaction and a summary of key findings^14, 15^. Interviews were recorded (with permission) and the audio file submitted to artificial intelligence voice recognition software (Otter.ai) to generate an initial transcription. |
| --- | --- |
| *Step 2: Familiarisation with the interview* | Interview transcripts were reviewed by LB to ensure they represented an accurate, verbatim account of the audio recording, and returned to each participant for the opportunity to amend their responses. |
| *Step 3: Coding* | Three researchers conducted open coding on a sample of transcripts *(n 2)* independently*,* to become familiar with contextual factors described by participants. Transcripts were coded against a draft analytical framework which closely reflected the CFIR including all constructs. |
| *Step 4: Developing a working analytical framework* | Informed by Step 3, the draft analytical framework was further refined by LB in consultation with the senior author. Both authors independently coded one transcript then discussed any discrepancies. The code names and their descriptions were reviewed and updated to ensure relevance to the data and study setting. |
| *Step 5: Applying an analytical framework* | All remaining interview transcripts were then coded by the lead author, line-by-line, against the refined analytical framework using NVivo qualitative data management software. The framework’s categories, codes and their definitions were iteratively updated *(Supplementary Material S4: Coding Framework)*. |
| *Step 6: Charting data into the framework matrix* | A matrix was created in Microsoft Excel to tabulate data from each participant relevant to the constructs and domains within the framework. Data coded to each construct was exported from NVivo and illustrative, verbatim quotes were charted systematically into the matrix. |
| *Step 7: Interpreting the data* | Data was interpreted via a two-fold thematic analysis approach.  Firstly analysing each construct independently by (i) reviewing the data charted for each construct within the matrix, (ii) identifying connections and commonly described concepts amongst participants, and (iii) articulating these as ‘sub-themes’ for each construct. A sub-set *(n 2) of constructs* were analysed using these three steps independently by two researchers and inconsistencies were discussed by all three authors. The data for remaining constructs were then analysed by the lead author.  The second thematic analysis served to identify cross-cutting themes by (i) reviewing the data across all constructs and domains within the matrix, and (ii) identifying factors which were described as either facilitating or impeding policy action by the City of Greater Bendigo. These cross-cutting themes were discussed by all authors and allocated to be facilitating factors, impeding factors or in some cases both, then visually presented to aid a succinct reporting of the results. |

## Additional Material A4: Coding Framework

Coding Framework informed by the Consolidated Framework for Implementation Research (figure adapted from Nolan & Warner, 2017^[[1]](#footnote-1)^)


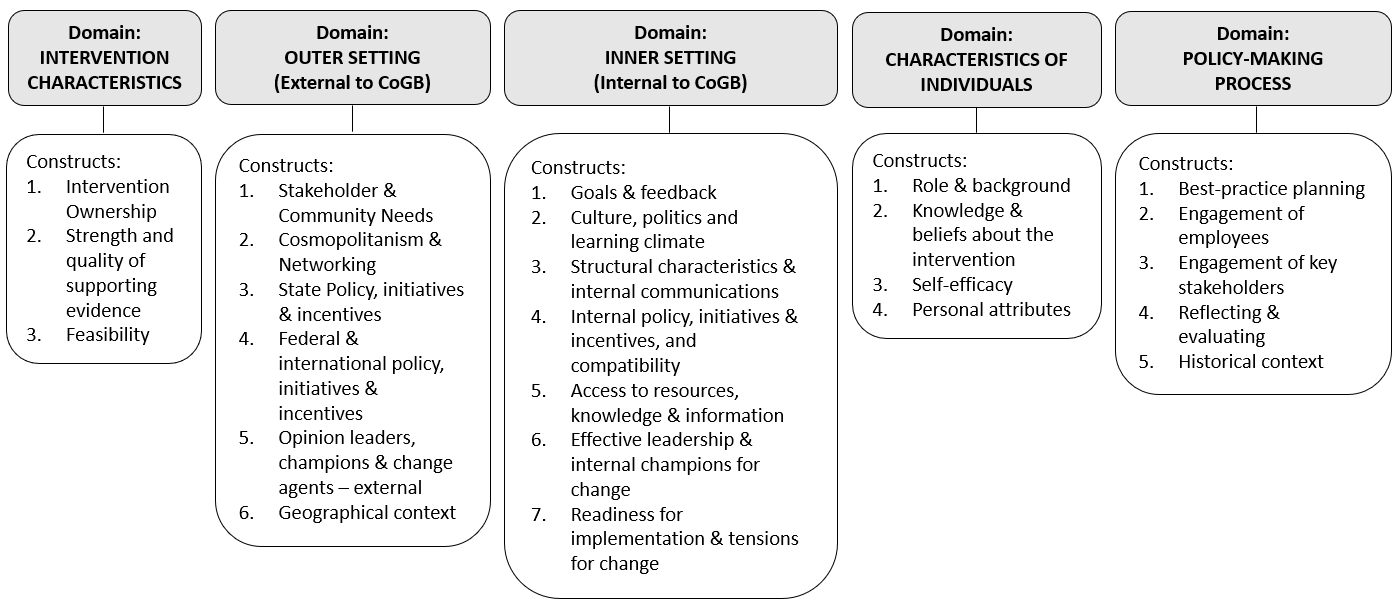


## Additional Material A5: Sub-themes and Examples of Illustrative Quotes for each Construct, organised by Domain

| *Domain* | **Intervention Characteristics** | |
| --- | --- | --- |
| *Construct* | Intervention Ownership | |
| *Description* | Perception of participants about whether the food policy is externally (beyond local government – community, state, federal, etc) or internally (within local government) driven/owned/developed | |
| *Sub-Themes* | | *Example of Illustrative quote(s)* |
| CoGB lead the development however the food systems policy is owned and implemented by many | | "Yeah, and we've got the services, not really state or federal, but the Regional Food Alliance is another not for profit organisation that we've got locally who I guess also advocate for local food as well." CoGB Employee, Project Officer  "I think local governments also have a big role to play in, like, collaboration, so getting agencies to work together. So, it might not be a policy that we're specifically developing, but we can facilitate conversations and collaborations to develop that may be looking at things like the infant program that influences dietary intake of first-time parents and that type of thing. So, helping with partnership and collaboration as well." CoGB Employee, Project Officer |
| CoGB have community’s best interests at heart and are trusted to lead food systems policy | | “Well, and I think it exists too because it's this is an area that's more important than egos, or that any one organisation is more important than another. Because it has the community's best interests at heart." External Stakeholder (Local), Senior Management |
| CoGB are well-positioned to drive collaborative efforts to develop and implement food systems policy | | "Oh, it's - no, local government. Local government, but supporting the whole system. We’re part of - Bendigo Health’s part of the local system. And we would be a small percentage of the work that that person would do, but it needs to connect... So there's so much connected work." External Stakeholder (Local), Senior Management |
| CoGB leads this work to support local residents and also smaller, neighbouring municipalities | | “"Bendigo is the biggest municipality in the region. And we do you know, we sort of play a bit of a big brother role supporting our neighbours. I guess it's just a recognition that, you know, people's lives aren't bound by municipal boundaries." CoGB Employee, Mid-Management |

| *Domain* | **Intervention Characteristics** | |
| --- | --- | --- |
| *Construct* | Strength and quality of supporting evidence | |
| *Description* | Participants’ perceptions of the quality and validity of evidence used to support the food policy, including references to evidence-based frameworks, knowledge and concepts applied in other settings. | |
| *Sub-Themes* | | *Example of Illustrative quote(s)* |
| Resources acquired are being directed to building up an evidence-base for future work | | "Yeah, absolutely. I'd also love a lot better local data. You know, we have had to invest hundreds of 1000s of dollars in the collection of local data that goes down to a granular enough level to inform our decision making across such a vast local government area. And we're lucky that we've been able to invest in that through our own funding and grants and stuff that we've collected. " CoGB Employee, Mid-Management  "The other thing we've got that the - it was money that we got through RDB is we're doing a social return on investment. For our organisations. That'll be really interesting too, once the results are ready or the recorder’s ready." External Stakeholder (Local), Senior Management  "But the thing is that we don't actually have a baseline so that in the next 10 years, we're not going to be sure if we don't set this up. We're not going to be sure - so that’s one of those things that we need to start thinking about now. We're not sure how many - we're not exactly sure how many different people are engaged with [inaudible] 36:31, for example. We're not exactly sure how many people [inaudible] 36:36 will identify with using sustainable agricultural practices. That's not the information that we're currently collecting. " CoGB Employee, Project Officer |
| Evidence created by CoGB is utilised by external partners to implement policy actions | | "Well, instead of having set up so many people growing food for Foodshare, there could be so many people, not being self-sufficient, but just being able to grow a little bit at home and look after themselves in that respect. It's hopefully that can transcend to then, them eating healthier, which means that Bendigo isn't, that 62% of Bendigo’s population is overweight and 37% obese. That'd be a nice change." External Stakeholder (Local), Mid-Management |
| CoGB partner with experts to create reputable data | | "So the other thing I failed to mention was in Healthy Together, they did the first Active Living census. So we also had data, which was fabulous. And so it's very hard to argue with the amount of data that we had, and it was done by - the whole census was done by the Social Research Centre. It was reputable data. And we've leveraged it within an inch of its life, really, and then used it to go and do more consultation and so forth. " CoGB Employee, Senior Management |
| Access to reputable data has informed CoGB’s prioritisation of policy options | | "But I suppose it's also driven by - Bendigo’s also had a bit of - some of the actual stats they get from the Active Living Census, the actual health indicators are a bit - and aren't really that impressive, you know, there's still -and that might be just another reason why there's been a great focus on health." CoGB Employee, Mid-Management  "It's about have you of leveraged it to grow seeds in areas where people experience food insecurity and have you provided opportunities for people to build the skills in how to actually grow. Have you provided free fruit and vegetables to people in those areas that our Active Living Census tells us have higher rates of food insecurity. So have you, I guess, embedded that lens of creating access across any opportunity, basically. " CoGB Employee, Mid-Management |

| *Domain* | **Intervention Characteristics** | | |
| --- | --- | --- | --- |
| *Construct* | Feasibility | | |
| *Description* | Participants’ perceptions about the relative cost/advantage, adaptability, trialability, complexity and the way the food policy is designed and presented publicly. | | |
| *Sub-Themes* | | *Example of Illustrative quote(s)* | |
| Participants describe food policy as a ‘fluid document’, acknowledging that the policy actions will change over time in response to community need and events (eg. COVID) | | | "The good thing about the Strategy is that it is a fluid document and things can be added when the need arises" CoGB Employee, Project Officer  "But at one point, he just said, “Ugh, I just - I'm not able to sell enough fresh things.” And so through the farmers’ market, they actually came up with these boxes. And that was a really great thing. Because the thing is that people - like these fresh herbs, people don't necessarily know what to do with them. They might get one, and then they like it and then they buy them and then they use a little bit of it, and then the thing is if they're not used to cooking with them, they don't use them to their full potential. And then they get rotten on the - they might rot on the kitchen sill and then that's it. They don’t use it, they don’t buy them again. But because they created these cooking boxes, it had three or four of his herbs, some pasta, got some pasta sauce, hams and cheeses, all sourced through the farmers’ market, those were sold out in two days and every week since. And it's really good." CoGB Employee, Project Officer |
| Participants are well-versed in the complexity of implementing food policy, with references to the need for a systems-thinking approach | | | "So we've recently made changes to our local planning scheme to make it easier for people who are buying into new developments to access fresh food. So for instance, there's now and I should double check that this has actually happened, because in the policy process, sometimes things that are said to happen, may not actually eventuate. But my understanding of what took place was that there's now sort of, I don't have the language, but like clauses in the planning scheme, that mean that major developments must happen within a certain distance of an Activity Centre, which is the place where sort of supermarkets and grocers exist, just from this sort of like a zoning perspective, if that makes sense. You've got your sort of little centres of shops with residential areas around that. And it's deliberately designed to prevent urban sprawl, and at the same time, protect agricultural land. But you know, the offshoot of that is that people live within walking distance of a supermarket or a place where they can buy fresh, healthy food." CoGB Employee, Mid-Management  "And regulation and legislation would be another in terms of reversing the food swamp phenomenon, and that needs to be complemented by a targeted approach to better access to healthy foods, nutritious foods and food literacy more broadly that is really reaching the people who are experiencing the burden of populational health most egregiously. Yeah, through a whole range of really well funded and resource campaigns that make good food affordable, and accessible and engaging and enjoyable for all ages. So, it's an integrated approach. But I think in terms of key policy initiatives, I think some kind of taxation measure on unhealthy foods and ultra-processed foods would be one. And reform of the planning framework to stop and reverse the food swamp phenomenon would be another." CoGB Employee, Project Officer |
| Participants describe the fine tightrope of balancing local resident and business needs with the health, environmental and economic outcomes they’re responsible for | | | "The largest family-owned chicken producers in Australia, one of. And then we've got a huge pork, intensive pork production. We still need protein. And we're with partly feeding the world in that, and partly feeding ourselves. How do we do that in a more sustainable way, working with them at a local based point of view? And then adding the plant side to that. So that's what I think we can do in recommending. But I don't know why, I think it's more cultural than anything else." CoGB Employee, Senior Management  "But in the move away from the amount of animal product we eat, cheap animal product, which is what it's coming from. That's a really fraught long-term proposition, I think, and for our environment. So if a council that was brave enough to have said, "We actually don't support that and that we actually support what's in our old environment policy, a humane food production system, that's novel. And so being able to actually see the benefits and find the actual investments that will come from other areas other than an industry that has a limited environmental benefit and health benefit for the community." CoGB Employee, Senior Management |
| CoGB is perceived by employees and external partners as being open to trial and pilot new policy actions to determine their feasibility | | | "Yeah, they were really conscious in Bendigo of - a lot of the pushback was around what should we provide instead if you want us to cut out the soft drinks and chocolates and chips and all that. So that was a really key part of why they did a bit of the trade display was to kind of show there's other options out there. And to kind of incorporate it into an event where it was kind of like a forum to share successes and to provide better training, and then have that all in one place." External Stakeholder (State), Mid-Management  "They just - they don't want council coming in and doing - and telling them what to do, or they just want to do what they want to do and don't kind of think about diverting from their core business. And they - yeah, especially things around healthy eating, they kind of can't see the need to. Yeah, they're the ones that aren't really doing much around healthy eating, that seems to be the case in those councils, whereas that's kind of the opposite in Bendigo; they're really willing to kind of try new things and anything that supports to help with their community, they're willing to kind of give it a go, so." External Stakeholder (State), Mid-Management |
| Limited resources and support from higher levels of government are considered to undermine the feasibility of effective execution | | | "our approach to food security and community involvement in food systems, that's very much part of the healthy eating story for the City of Gastronomy work. So you end up with these mutually enforcing areas of work, which further legitimise us being in that space. But having said that, there's not a lot of resources" CoGB Employee, Senior Management  “Yeah well that's the economic story that has to be told. So that public health doesn't just get to this little fringe of you know healthy eating, it has to be seen as an economic force that's a viable business option." External Stakeholder (State), Senior Management |

| *Domain* | **Outer Setting** | |
| --- | --- | --- |
| *Construct* | Stakeholder and community needs | |
| *Description* | The extent to which stakeholder needs, as well as barriers and facilitators to meet those needs, are accurately known and prioritised by the local government. Reference to the local government’s interest or concern for community need during the development of the food policy. | |
| *Sub-Themes* | | *Example of Illustrative quote(s)* |
| CoGB have created networks, platforms and other opportunities to understand stakeholder and community needs. | | "I think I think that the process of developing the food system strategy itself and the way that Bec and Vicky and others sort of assembled a working group has aided that in that it had people from our resource recovery area, it had people that will work on our environment strategy… The vision itself was actually entirely developed by a group of community members through essentially a citizen’s jury process. So there was a basically a third party, like an organisation called xxx that helped to recruit sort of a random stratified representation of the community, and we did all sorts of briefing materials for them that built on earlier engagement that we'd done… But they'll be sort of impatient to see things change and some of their issues probably taken up. " CoGB Employee, Mid-Management  "And having that agricultural voice is really, really important. Because it's probably a group that is not commonly engaged. It's probably not easily engaged with, unless you actively go and seek it out." CoGB Employee, Project Officer |
| CoGB are legally and morally required to listen to and respond to community need | | "To my way of thinking, they're there to listen to what the community has to say to help them rather than, this is how we'll help the community, that type of thing. And I think that that can be dangerous on a number of levels, but particularly for wasting resources and duplicating things" External Stakeholder (Local), Senior Management  "So I think the challenge is breaking through with people who don't necessarily know or have the time or think the way to understand the whole scheme of it, you know all the bits that join together, that systems thinking of how it all works. So that's the nut that needs to be cracked." External Stakeholder (Local), Senior Management |
| CoGB prioritise policy and other activities in response to the needs of their community | | "So it's more - as I said, our whole focus as a local government is actually community wellbeing. So if community really is demanding this and we build that momentum, then that's what we'll be focusing our resources on, making sure that that continues that way. So whether it's economic development for small producers or large producers locally, that's what we'd be spending it on, rather than at the moment we're probably spending more on supporting manufacturers of various other products. So it's really - it's sort of trying to sort of change that community mindset so that that then drives the way that their rates are paid, their rates are spent." CoGB Employee, Senior Management |
| Participants acknowledge a variety of barriers involved in meeting some stakeholder needs and expectations of CoGB. | | " I think our community in general is quite conservative. And we try our very best to navigate and respect that. I think the traditional meat and three veg is certainly a space that we still dwell in as a community. And I think it's local governments respect to our community, you know, that's a cultural norm in our location, but it's probably not territory that we were too game to sort of break into just yet." CoGB Employee, Mid-Management  "So we are a political organisation. And so, we would have built enough community momentum that this is exactly what they want. And so as a result, our whole system works around community need, and so that communities can actually exercise - you know, can choose a healthy option. Healthy, sustainable option, regardless of where they live, and what their sort of socioeconomic background is. So for me, it would be about we’re allowing people the option that - the options to buy locally, to produce locally, to eat healthy food, to have access to things that - so to remove all the barriers, really, that's what we'd be having in place, I reckon." CoGB Employee, Senior Management |

| *Domain* | **Outer Setting** | |
| --- | --- | --- |
| *Construct* | Cosmopolitanism and networking | |
| *Description* | The degree to which the local government is networked with external organizations as part of a broader, equal, inclusive community – other local governments, community groups, state government, etc. | |
| *Sub-Themes* | | *Example of Illustrative quote(s)* |
| CoGB plays a leadership & coordinating role | | “We've led the way with some of our own policies. So a lot of our work, we start by looking at our own practice first. So whether it's a safe, healthy food and catering policy. It’s a bit rich to go out and ask someone else to do something if you're not doing it yourself.” CoGB Employee, Senior Management  "I guess just showing what's possible, particularly in the Australian context, with the food system strategy, yet it's also I guess about leadership and taking on that coordinating role when looking at our local food system, and that's primarily just across the LGA. There's a lot of other small local government areas around us that we are happy to support, you know, as best we can." CoGB Employee, Project Officer |
| CoGB shares what they’ve learnt from working closely with their community with networks beyond their local government | | "Yeah it doesn't seem to be recognition, like they don't seem to want to be promoting themselves too much. They’re a bit more humble. I think it’s more – yeah I think it's more just them wanting to really share what they've learnt and really contribute to that bigger public health kind of work in Victoria rather than just their little bubble in their area. And yeah I think it probably comes down to probably the staff, the people and their personalities. I think that they kind of are willing to be involved in that kind of system rather than just focused on their own work and what their role requires of them and that sort of thing." External Stakeholder (State), Mid-Management |
| CoGB's commitment to attracting grants and awards at the state and international levels connects them with diverse and influential stakeholders and networks | | "So just someone who dropped in from UNESCO, from Sweden and the guy said oh, this is what we're doing and, and through a contact of the current Mayor's. So we found out about it. And then we found that there was a process that you could actually do to apply. And we were doing our food systems strategy at the time and thought, well, that's a good idea." and " But then we looked into it and as we talked to the guys from Sweden, who runs their programme, and people from Parma, in Italy, and the network of 53 across the world, we thought actually, it's not about high, high level eating, that we thought it was either. It's actually about providing equilibrium in the food system. As well as the high end food and wine and everything else. Yes, we've got that. But it's more about understanding the food system, and applying a more resilient and sustainable food system into the future." and "One is that we sit on the international group that sits across, chaired by a guy in Sweden, the Swedish government provides the auspice for that." CoGB Employee, Senior Management |
| CoGB values reciprocity, whereby they actively contribute to networks while also gaining a lot in return | | "our environmental health team have really strong connections with other local governments through their networks." CoGB Employee, Mid-Management  "respecting Indigenous knowledges - the Djadjawurrung were heavily involved in that process, and are heavily involved in a lot of the actions that are coming out of that. So, that's just fantastic for Bendigo and the wider region, I think." External Stakeholder (Local), Mid-Management |
| CoGB benefits greatly from their cosmopolitan efforts including greater capacity to meet specific community needs, attracting funding and broader recognition and recruiting and retaining a skilled workforce | | "I think there's nine local governments that are involved in that. Because it's seen as being a region of gastronomy rather than just a city" CoGB Employee, Project Officer  "the healthy eating advisory service, and VicHealth are probably the two, the biggest kind of drivers for that work and supporters were working in that space. And that's funding but also resources as well to kind of support it. And then probably the education settings as well side, I think it was xxx for that one who did the evaluation side of the world to show what works, how does it actually impact these businesses" and "it's flowed on to a healthy sports rewards project as well. There's been kind of a community of practice with the pilot councils. So Frankston and Shepperton are two who spring to mind, and there's a couple of others. I think it was five councils to do the water and sport project" CoGB Employee, Mid-Management |

| *Domain* | **Outer Setting** | |
| --- | --- | --- |
| *Construct* | State-level policy, initiatives & incentives | |
| *Description* | The influence of external strategies (both past and present) to prompt food policy action | |
| *Sub-Themes* | | *Example of Illustrative quote(s)* |
| Mandated influence, whereby state-level policies and regulations required involuntary action by the CoGB e.g. Municipal Public Health and Wellbeing Plan, Local Government Act | | “the main legislative driver of local government action initiatives in this area in Victoria, which is the 2008 Public Health and Wellbeing Act. Which of course, made it a legislative obligation for councils to develop Public Municipal Health and Wellbeing plans. Which, in some instances, not everywhere, but in some instances, and increasingly so, is flowing through to food systems and food policy” CoGB Employee, Project Officer  “Certainly, look, it would certainly be limiting the intake of ultra-processed nutrient poor over packaged foods. And, and avoiding over consumption beyond caloric requirements. Ultimately, because the Victorian government's public health and wellbeing plan includes those elements in its healthy ageing section, which is an action area of the public health and wellbeing plan. Consequently, councils must write their own public health and wellbeing plan as well and have you report on anything that is in the Victorian plan. So as a consequence, because state government publish that as an action area, it becomes one in ours as well. But it's certainly close to the heart of a lot of people that work within our units as well.” CoGB Employee, Mid-Management |
| Supportive influence, whereby state-level interventions facilitated the CoGB to progress their own, locally relevant food sustainability agenda e.g. Hospital Procurement Victoria, VicHealth’s Water in Sport, Healthy Together Victoria, Healthy Choices Guidelines, Victorian Population Health Survey collection, Nutrition Australia’s Healthy Eating Advisory Service, Cancer Council’s Achievement Program, INFANT, Healthy Food Connect | | “One of the areas that I think maybe is fairly unique, and I think is really exciting, is we've got a newly adopted Itinerant Trading Policy. So itinerant trade is like, I don't know, your donut vans and coffee vans and stuff that goes on, you know, they park at your local parks and, and sell stuff to you. So those are, those are for profit businesses that are using community land. And you know, they're effectively getting free rent, whereas other businesses are having to pay for rent. So to sort of level the playing field, our council recently implemented an itinerant trading policy that required them to pay some fees. But as part of that, we introduced an incentive so that any food itinerant traders, who were selling their food, according to Healthy Choices Guidelines got a significant discount on those fees. So I think that that's a really innovative kind of approach to have taken to see that there was that lever available and to pull it.” CoGB Employee, Mid-Management  “We've seen it in our street vendors, Healthy Choices written into that policy as well. And we also, I think there's a role to be played in planning and advocacy. I'm not really well versed in that, it's not my area of expertise. But even things like they are rating and revenue review, advocating back to the state government for changes to the rating system that allows us to, I guess, provide discounted rates for venues that provide Healthy Choices as a way of being able to support a change in the community.” CoGB Employee, Mid-Management  “So I guess at the state level, they've provided the funding for Healthy Heart of Victoria. Because it was initiated regionally and has this place-based angle, state government hasn't been as hands-on in determining where and what we’re going to focus on for this part of the phase. So funding, yes, but then it was up to each LGA to determine where that where they wanted to invest infrastructure and activation funds, we called it.” CoGB Employee, Mid-Management |
| Prohibitive influence, whereby state-level policy impedes CoGB’s efforts to progress the food sustainability agenda e.g. Class 4 Simple Sausage Sizzle regulation | | “I've talked about that letter of political opportunity, you got the politics at the top and the community at the bottom. The community can entertain and be part of the agenda setting. But without the resourcing and the policy settings, you just can't get the outcome.” CoGB Employee, Senior Management  “what department of health calls Simple Sausage Sizzle. It's a very fine scope of what's permitted under there, being sausages, bread, sauce, and onions. And cake stalls is another example of fundraising activities that require no regulatory involvement really, no fees or anything like that. Most of the things that are considered to be safe and low risk and promoted as fundraiser activities are the type of food that perhaprs may be leading moreso to chronic health issues when they're consumed on mass. That's a very, very long way to get down to the point I'm trying to make. But I guess what I'm trying to say that the Food Act is certainly working against the type of healthy eating messages that we're trying to get out there into our community by making the perhaps the unhealthy choice, the easiest choice.” CoGB Employee, Mid-Management |
| The state governments Health and Wellbeing Act and Local Government Act has progressed to reflect planetary health evidence | | “So the guidance from the state government, I suppose, comes - you can read it in a lot of ways, but it comes from the way local government's role is described as being about wellbeing. There's some overarching governance principles around the environmental, social, and economic outcomes for the community, but it also talks about - and it also talks about future generations now.” CoGB Employee, Mid-Management |
| Where action and alignment between international, federal, state and local policy action is missing, CoGB fills the gap and/or advocates for change | | “Particularly the regional cities is what we were sort of saying, “Well, look. We've gone ahead and we've got this international recognition for you, Victoria, so you should support us.” And that advocacy works. They've actually put some money in the new creative industries strategy for the state to support the creative cities of Victoria, so.” CoGB Employee, Project Officer |

| *Domain* | **Outer Setting** | |
| --- | --- | --- |
| *Construct* | Federal and International policy, initiatives & incentives | |
| *Description* | The influence of external strategies (both past and present) to prompt food policy action | |
| *Sub-Themes* | | *Example of Illustrative quote(s)* |
| Global recognition via UNESCO's Creative Cities Network prompted the documentation of existing good practice and aspirational targets | | “I think it's provided a series of attention to what we've done. , We know that we've actually attracted new business around that. We know, that's actually the case where a couple of restaurants have moved here since. And there's a whole heap of other things that have, from the ground, that have been made more visual. So a lot of things that were happening before at a lot lower level, now we've now got a platform for them to actually communicate it and so that's been really good. We were already on the path around the food policy stuff. So it's probably an added value. So we had the food system strategy. And thinking about that, I think what the Gastronomy stuff does is really give us another and a higher level order, sort of audience, but also authorising environment to really push things. And that's where we want to sort of take it next.” CoGB Employee, Senior Management |
| Where action and alignment between international, federal, state and local policy action is missing, CoGB fills the gap and/or advocates for change | | “I think there's traditionally always been some Federal level leaders, but from a from a sustainability waste angle, that have been fairly lacking in the last decade, for reasons I won't get off track on this conversation about. However, I think there's been thinking and focus that at a State level to try and support it, but at the end of the day, it's really coming down to, at least in the last five years and probably for the next couple of years, down to local government, with teams and people that have either a mix of the right expertise, but more importantly, just the drive to try and get something like this over the line.” CoGB Employee, Mid-Management |
| City of Gastronomy application prompted new partnerships (eg. international stakeholders) and strengthened existing partnerships (Djadjawurrung) | | “But then we looked into it and as we talked to the guys from Sweden, who runs their programme, and people from Parma, in Italy, and the network of 53 across the world, we thought actually, it's not about high, high level eating, that we thought it was either. It's actually about providing equilibrium in the food system. As well as the high-end food and wine and everything else. Yes, we've got that. But it's more about understanding the food system, and applying a more resilient and sustainable food system into the future.” CoGB Employee, Senior Management  “Yeah, so even before we were successful in our designation, we engaged with quite a few of the other creative cities of gastronomy, particularly with Östersund in Sweden and with San Antonio and Tucson in the US. And a couple of the Italian cities of gastronomy.” CoGB Employee, Project Officer |
| The City of Gastronomy designation has strengthened food system action, while simultaneously driving health, tourism and economic outcomes for the community | | “I would like to think it was the health and the social connection, but that's because I come from community. But I would say that what would have tipped them over would have been that City of Gastronomy pitch. And the economic drivers into tourism behind it would have been the, oh, well, we can justify putting some resources and spending money in this space because we get all of this return, I suppose, out of it, and the return is always about the dollars. It's not about the social capital necessarily in organisations like LGAs.” External Stakeholder (Local), Senior Management  “I guess having council endorse those recommendations and carry them forward meant that we had that authorising environment to go forth. So, certainly, it can't be with our council. A lot's been driven from the health and wellbeing area, but it's right across to our sort of waste services, economic development, sustainability. Tourism, with the city of gastronomy. Environment. I think it hits all areas of our council, really, to some degree.” CoGB Employee, Mid-Management |
| CoGB is motivated by a moral responsibility to contribute to international and federal targets | | “We're linking all of our work too to the Sustainable Development Goals. So we were using One Planet Living, but we've moved a bit more to sustainable development goals because we're part of the larger world.” CoGB Employee, Senior Management  “And why are we in front? Because we have leadership. We've identified a problem, and without, to be honest, without the support of any federal real federal statement we've just gone and said we need to do something about it, because you're not.” CoGB Employee, Senior Management |

| *Domain* | **Outer Setting** | |
| --- | --- | --- |
| *Construct* | Opinion leaders, champions & change agents – External | |
| *Description* | Reference to the influence of individual people (non-employees of the local government) to prompt food policy action. | |
| *Sub-Themes* | | *Example of Illustrative quote(s)* |
| Passionate individuals from the community and partner organisations enable CoGB to turn ideas into action | | “Some really key people and, like, xxx from xxx, a very huge fresh-food, local-food advocate because that's what her restaurant is based on. She's also very passionate about those businesses. So, like, a farmers’ market, it’s got that same sort of thing. There was a food fossickers' group, which is kind of around the tourism space; and then, of course, there was, like, xxx and other community gardens, a few of the Stephanie Alexander things. So, there's a lot of stuff going on that - excuse me - is slowly being linked through some of those projects. So, just a different way of doing it rather than sitting back and saying, "let's just do a policy".” External Stakeholder (Local), Senior Management |

| *Domain* | **Outer Setting** | |
| --- | --- | --- |
| *Construct* | Geographical context | |
| *Description* | References to the positioning and infrastructure in relation to their impact on effective policy-making | |
| *Sub-Themes* | | *Example of Illustrative quote(s)* |
| Bendigo’s size and distance from Melbourne are conducive to food sustainability policy action | | “There's a historic advantage with the City of Greater Bendigo that it's, since management of resources, so dealing with household waste and that sort of thing, has been in house predominantly. So, because the City of Greater Bendigo technically being today, a regional city, is almost like a miniaturised version of Melbourne as a whole. So, it has to be relatively self-sufficient, versus I guess, if you're a council in a metropolitan area, you have things that actually just sit in other councils more predominantly. And so, there's been the historic infrastructure here that the council runs its own landfill and has to manage a lot of that. And as part of that, that's part of the, I guess, fee that commercial operators pay for depositing their waste in a hole in the ground at the landfill. There's overtime, been built up, I guess some savings as sort of a waste fund at the city. And when that was combined with access to some State level funding, was able to then roll out the infrastructure and having those green bins around the region and then getting that educational aspect in play as well.” CoGB Employee, Mid-Management |

**DOMAIN: Process**

| *Domain* | **Process** | |
| --- | --- | --- |
| *Construct* | Best-practice Planning | |
| *Description* | The degree to which the activities / strategies within the food policy have been developed in advance and based on best-practice evidence | |
| *Sub-Themes* | | *Example of Illustrative quote(s)* |
| The policy-making within CoGB is known to be complex and time-consuming, allowing for careful planning and preparation of evidence to support the process | | “I mapped out - initially, I did a literature review of what local government areas have worked in this space and who had gone through a similar process. And I spoke with Cardinia Shire Council. So, they had recently had their community Food Strategy endorsed, and so I sought their advice on the process that they went through, and I know that Sustain were heavily involved in their development process and still are in the implementation. So, that's sort of why I contacted Sustain and just knowing that they're leaders in this space and national - a national agency organisation. I just thought it would add a lot of value to the process. And also, speaking to other local government areas as well. I know they had developed strategies, but a lot of them had developed, like, just relevant policies and things. So, yeah, and just looking at best practice for community consultation in general as well helped inform, yeah, the engagement plan, I suppose.” CoGB Employee, Project Officer |
| Progressing the sustainable food policy action required dedicated human resources, which eventuated after effectively advocating to elected council representatives with the use of evidence | | “I thought, well, how are we going to get these community gardens? And I was told, well, there's no food policy. It doesn't sit anywhere. I'm like, what do you need for that? You need to have resources. We've got no staff. We can't do that. So how do you go about getting the staff? You got to get a budget bid up for the budget cycle, so you can employ someone to look at the work that you need to engage the community to get a draft, all that. So I worked on that and one of the ways of elevating that, which is a slightly separate way of influencing was to at the council table with the managers and the CEO, to say to the CEO, "You're not fulfilling your obligations under the Local Government Act for healthy eating currently. What are you going to do about that?" CoGB Employee, Senior Management  “That actually had quite an impact. And so then I advocated around the table that we needed a budget bid for a position for a healthy food policy or a food policy officer. I advocated for several roles, but that one got up.” CoGB Employee, Senior Management |
| Planning for effective policy-making at the local government level requires advocacy and change to occur at higher levels of government | | “I mean we've often argued for the State government to make significant changes to their state planning policy, or the planning scheme. It's probably the most pivotal piece of state government legislation that affects local government ability to make change in the food environment… their planning scheme is helping and hindering local communities' ability to create healthy food environments and healthy and sustainable food environments. And there's a lot of levers that they've got within that.” CoGB Employee, Mid-Management |
| CoGB called upon experts to inform and facilitate the planning stages of the food policy. | | “kickstarting the initial three-month consultation period - we ran a workshop - it was called 'forum' - that we hired Sustain to come down or facilitate and to also present on the work that they're doing. And that was also mapping what Greater Bendigo's key issues and opportunities were… And we were lucky with - there was a lot of interest, and we got representatives from across the food system attending.” CoGB Employee, Mid-Management  “And there's a handful of other councils where we've got good relationships with them where we might tap them on the shoulder and be like, “Hey, can you review this for us?” or “Can you tell us if you think this will work?” or that sort of thing, and they are always willing to kind of give us their feedback, because they know that it will help others, to get them on board too, so.” CoGB Employee, Project Officer |
| CoGB has a history of integrating credible frameworks, data and evidence-based theory into their work | | “We've had a very good environment strategy, that was before I got on to council, which reflected the One Planet Living Principles, if you'd be well aware of those.” CoGB Employee, Senior Management  “Certainly, local data has been a really critical driver of our choices. Data that's provided by a whole range of sources but also data that we've collected ourselves.” CoGB Employee, Mid-Management |

| *Domain* | **Process** | |
| --- | --- | --- |
| *Construct* | Engagement of Employees | |
| *Description* | Extent of engagement amongst local government employees who have been formally appointed with responsibility for implementing the food policy. | |
| *Sub-Themes* | | *Example of Illustrative quote(s)* |
| CoGB employees involved in the food policy are passionate about improving their local community | | “I think that most of the motivation comes from the city and they wanting a healthier community. So I think that the success of the City of Greater Bendigo has been because of the staff, the employees of our local council. They're innovative. Their goodwill towards their community, their motivation and drive to make Bendigo a better place I think mostly comes from them outward.” External Stakeholder (Local), Mid-Management  “Yeah, I think there's pockets where there's really amazing work happening and really genuine relationships being developed, and there's probably other areas where it's still seen as just another hurdle to overcome to do what you've always done.” CoGB Employee, Mid-Management |
| CoGB fosters an internal culture of innovation, recognition and boldness. | | “And I guess that is the challenge that we face is that local governments being very bureaucratic in nature, quite often stifling innovation by all of this risk aversion that seems to come naturally into our environment.” CoGB Employee, Mid-Management  “There's definitely a lot of goodwill. And I've just been working today on an application to Djadjawurrung around possibly dual naming of the new council plan to reflect that as well. Because that's what the councillors wanted. But you know, it's - I think City of Greater Bendigo as an organisation is 1,000 odd staff.” CoGB Employee, Mid-Management |

| *Domain* | **Process** | |
| --- | --- | --- |
| *Construct* | Engagement of Key Stakeholders | |
| *Description* | Extent to which key stakeholders have been involved in the planning and implementation of the food policy e.g. community consultations, representation on City working groups | |
| *Sub-Themes* | | *Example of Illustrative quote(s)* |
| CoGB exceeds mandated expectations to engage stakeholders in core business (e.g. Municipal Public Health and Wellbeing Plan) | | “The food system strategy, the engagement for that was really something else. It was very professional and there was up to 1,000 people consulted in different ways. It was a great community engagement process and the materials used for that were really professional, properly printed. This is the resources that local government brings to something. If we've gone out as a food alliance and said we want to talk to community about policy, we would have had a few meetings and dinners and all that and put together some notes and made a little whatever. It would never have the status that you would get immediately with local government.” CoGB Employee, Senior Management |
| CoGB has invested in the establishment and facilitation of a number of advisory and working groups, each with a different role to play within the food system strategy | | “But the fact that it's, I guess, part of our, our planning and everything now as one of the requirements. Yeah, so definitely a partnership model. We can't do it on our own, right? Yeah. And they have different parts of the community that they interact with, and a different role to play. So, partnerships are a huge part of our work.” CoGB Employee, Project Officer |
| CoGB engages community at the grassroots level and participants acknowledge their leadership role in facilitating the journey from where they’re at to achieving systemic change | | “They actually spent a lot of personable time talking to the organisations that could help them to fulfil these things, or to people directly, like I said at the markets or organisations, or people involved already in schools with Stephanie Alexander Gardens, and all of that sort of stuff. So, they reached out into the community at a more grassroots level or with organisations that had a more grassroots level to give them the information.” External Stakeholder (Local), Senior Management |
| CoGB values community engagement because it informs policy prioritisation, leads to smoother execution and reduces unnecessary duplication of work. | | “I guess the other side of it really is coming back to first principles of community ownership as well, which local government is very much about, well we say we're about that, in our current state. We say we're about local community members as part of the decision-making process but realistically, policy procedure legislation means that they always a couple of steps away. So I think community engagement right at the core of local government, in their decision making, would lead to a utopian state of community ownership.” CoGB Employee, Mid-Management |
| COVID affected attendance and representation at these established groups, particularly primary producers, which reinforces the importance of investment in face-to-face engagement pre-COVID | | “it used to be the first one there was at least 100 people… Yeah, a huge interest… But then probably, since COVID. it could be up to 25 to 30 people.” External Stakeholder (Local), Mid-Management  “The first couple of meetings around that the City of Greater Bendigo held were attended by, like, 80 to over 100 people. A lot of those people dropped off quickly, and then it's like really dwindled down for many reasons. COVID obviously hasn't helped that either.” External Stakeholder (Local), Senior Management |
| The size of CoGB and the personalities of CoGB employees facilitate effective community engagement. | | “It could potentially come down to I think that the staff that they've had at Bendigo Council have been really proactive in a lot of their community engagement in terms of them going out and working really closely with those organisations, and yeah, whether it's just like the personalities of those particular staff members that are really engaging and can get them on board really well. And maybe – yeah, my sense is that it's just that sense of community in the in the Bendigo area; they seem to have be quite a tight knit community that they're quite supportive of these types of things.” External Stakeholder (State), Mid-Management |

| *Domain* | **Process** | |
| --- | --- | --- |
| *Construct* | Reflecting and evaluating | |
| *Description* | Quantitative and qualitative feedback about the progress and quality of implementation. Reference to monitoring, evaluation and reporting processes | |
| *Sub-Themes* | | *Example of Illustrative quote(s)* |
| When prompted to reflect on their progress, CoGB employees are proud yet don’t consider their work exceptional | | “Not to be cynical but it's interesting when you used the word trailblazing because sometimes I think it's also, that it's not so much that you're that far out ahead but the others are so far behind.” CoGB Employee, Senior Management  “Yeah, and my reflection may not be - it may not be fair or whatever, but I've come around to the view that Bendigo writes some beautiful strategies, but it's not necessarily always - like the implementation often doesn't quite reflect the beauty of the aspiration.” CoGB Employee, Mid-Management  “Lots of good work happening, too. But I guess, again, the closer you get to it, the more you realise the gap between aspiration and reality, I suppose. And the follow-through, sometimes.” CoGB Employee, Mid-Management |
| When prompted to reflect on whether food sustainability should be addressed by local government, participants considered it inextricably intertwined with health, and the logical way forward. | | “I guess when people first started having to wear seatbelts it was annoying. It was an invasion of privacy. Well, you gave it a few years, and it just became the norm. Like Point-O-five became the norm. So many of these things when they get introduced become the norm. So why can't they become something along the line of that food insecurity, or that sustainability, because if everyone's doing it in a few years, it just becomes what is, and well – I guess I see, the only way something can come sustainable is with manpower.” External Stakeholder (Local), Mid-Management |
| When prompted to reflect upon why CoGB has invested in food sustainability policy, participants described Bendigo as attracting a skilled workforce who value social connectedness, mental health and wellbeing. | | “Bendigo is a real cauldron of passionate people who they've had their experience in Melbourne, I guess, and then they've come to Bendigo for, I don't know, the beautifulness of it all. Really skilled, passionate people. …. And its location within Victoria I think is ideal. We're a little bit bougie, you know, here in Bendigo. People want that lifestyle.” External Stakeholder (Local), Senior Management  “And majority of people had gardens, they're out in their garden. They do incidental activities. So they're not using leaf blowers, they're using a broom, or they ride to work, they walk to the shops, that type of stuff. They have downtime, they drink wine, they drink coffee, they have fun, they have friends, they have social outlets, okay. That's what I grew up in as a child. And these are the elements of social connectedness and mental health and wellbeing. It's all connected. And so, going back to the farmers’ market, there’s that whole social connection and interaction at the farmers’ market - getting your food - and that is really what interests me. I'm really interested in a society and a community that goes back to that and gets us out of those big supermarkets, because they've gotten our souls” External Stakeholder (Local), Senior Management |
| CoGB employees don’t consider themselves well-equipped to plan and execute monitoring and evaluation processes | | “So, I'm sure someone would have mentioned the one planet living framework that we've adopted within Council, and we've utilised it in the Strategy as well. I think the issues that we've come up against is measuring this. So, how is it measured, and how do we do that at a local level?” CoGB Employee, Project Officer |

| *Domain* | **Process** | |
| --- | --- | --- |
| *Construct* | Historical Context | |
| *Description* | Reference to policy and practice that has happened in the past, which is considered relevant (either in facilitating or impeding) current policy action. Also includes references to perseverance over time. | |
| *Sub-Themes* | | *Example of Illustrative quote(s)* |
| CoGB’s historical focus on food insecurity as a public health issue led to addressing the food system challenges more broadly as state and federal priorities shifted. | | “I think there's reasons why they've been active in food policy and food systems, which I think go back to a kind of a policy orientation in this area, that probably even predates what I would nominate as the main driver, the main legislative driver of local government action initiatives in this area in Victoria, which is the 2008 Public Health and Wellbeing Act. Which of course, made it a legislative obligation for councils to develop Public Municipal Health and Wellbeing plans. Which, in some instances, not everywhere, but in some instances, and increasingly so, is flowing through to food systems and food policy. So, they were involved in an earlier initiative, which is the Food for All initiative, the food security focus that I think, again, may have had linkages with the Victorian Local Governance Association, and I believe with VicHealth in terms of a funding partner.” CoGB Employee, Project Officer |
| Changes in state and federal politics require local government to pivot their policy agendas | | “Victorian Food Network - I couldn't always get the words right. It was such a long time ago. That was around the time where politically there was interest in food policy at a state level… they were doing ministerial level, looking at advisory groups around food and food policy, and even departments, dedicated departments in the government. There was a whole lot of interest in work starting to happen. And then politics changed because the party in power changed. And it all went out the window.” CoGB Employee, Senior Management |
| CoGB’s food sustainability policy is the result of many years of perseverance, and reflects a mature narrative | | “The fact that they can equally have a conversation around the importance of the food systems work and healthy choices guidelines, and you know, you can just create - and this is the difference between, I guess, how leadership operates. Sitting around the table - in Mildura, I talk with the then equivalent on the development of a new recreation centre or something, and I go, “How about an air fryer?” then they’d go, “Oh, oh, oh, oh, oh.” Compared to down here, “How about an air fryer?” “Oh, yeah. Already thought about it.” That's the difference. The conversation is more progressive… it's just I guess the maturity of the conversation here in Bendigo. It's mature.” External Stakeholder (Local), Senior Management |
| Participants describe a number of pivotal events in politics as influencing their current food sustainability policy; for example; International: UNESCO’s Cityy of Gastronomy Designation, Sustainable Development Goals. Federal: National Preventative Healthy Taskforce, People’s Food Plan.  State: Healthy Together Victoria, Food for All Initiative, Public Health and Wellbeing Act, Water in Sport (VicHealth). Local: Declaring a Climate Emergency, Healthy Heart of Victoria. | | “I think there's reasons why they've been active in food policy and food systems, which I think go back to a kind of a policy orientation in this area, that probably even predates what I would nominate as the main driver, the main legislative driver of local government action initiatives in this area in Victoria, which is the 2008 Public Health and Wellbeing Act. Which of course, made it a legislative obligation for councils to develop Public Municipal Health and Wellbeing plans. Which, in some instances, not everywhere, but in some instances, and increasingly so, is flowing through to food systems and food policy. So, they were involved in an earlier initiative, which is the Food for All initiative, the food security focus that I think, again, may have had linkages with the Victorian Local Governance Association, and I believe with VicHealth in terms of a funding partner. So, that goes back as far as I'm aware, back to 2005. And I think Bendigo were one of the councils that were involved in that. So, there was some years of work that was done at the local government level from that Food for All focus. And then, in the wake of that, there was the federal action on the health prevention in the time that xxx was the Federal Minister of Health with the focus on prevention and the National Preventative Healthcare Taskforce. And the funding that flowed from that at the State government level in Victoria became the Healthy Together Victoria programme and initiative.” CoGB Employee, Project Officer |

| *Domain* | **Characteristics of Individuals** | |
| --- | --- | --- |
| *Construct* | Role & background | |
| *Description* | Reference to the position held by an individual and/or their professional or personal background and how this influences their work relevant to the food policy. | |
| *Sub-Themes* | | *Example of illustrative quote(s)* |
| Participants bring a breadth of knowledge and experience to their work on the food sustainability policy, spanning areas of health, nutrition and dietetics, food safety regulation, social planning, public policy, circular economy, food rescue logistics, management and leadership (eg. CEO level), sustainable development, academia (eg. three participants commenced a PhD) agribusiness, water management and community development. | | N/A |
| Participants describe their professional experience aligning with personal motivations for health and sustainability outcomes | | “I used to as a really little kid cook with my nan. Which is probably what got me really interested in food was really through cooking. And sustainability wise, I feel like mom's always been interested in it too. As soon as we could start recycling, we were doing it. … Which I guess I'm able to draw on what I've learned over my years of working as a dietitian. And I guess a large part of this role is kind of policy focused.” CoGB Employee, Project Officer |
| Participants consider their own professional experience as one part, or complementary, to the collective expertise of CoGBs workforce | | “I haven't done a Master's of Public Health or anything like that, sort of more of that human rights lens and social justice lens, from my training across. And that seems to be a pretty good - it aligns pretty well, I’d say, and there's a lot of people around here with more sort of, I suppose, specific expertise in terms of nutrition and diet. But helping to sort of try to translate that a bit into policy and advocacy has been - I think it's sort of worked really well.” CoGB Employee, Mid-Management |
| Participants with professional experience beyond local government describe a steep learning curve regarding the reality of policy-making processes | | “So it's all been quite a learning curve about how decisions are made and how it all works and how much council does. I think people often underestimate what local government does, like it has just so many, so many different areas that it has influence in, and I didn't know that before I started working at local government.” CoGB Employee, Project Officer |

| *Domain* | **Characteristics of Individuals** | |
| --- | --- | --- |
| *Construct* | Knowledge & beliefs about the intervention | |
| *Description* | Participants’ attitudes toward and value placed on the food policy, as well as familiarity with the evidence and principles underpinning the food policy | |
| *Sub-Themes* | | *Example of Illustrative quote(s)* |
| Participants describe health and environmental sustainability as being inextricably linked | | “I think if you're thinking about health, and not thinking about environmental impacts, you're not really taking into consideration the full picture of health. Yeah. Because the effects that we're having on the environment are also likely impacting on human health as well.” CoGB Employee, Project Officer |
| Participants articulated many reasons why experts are calling for an urgent transformation of our current food system | | “Oh, simply it's because we're overusing the resources that we have on the planet, in a macro sense. And in a micro sense the diets themselves, if you look at the whole global population aren't adequate to sustain parts of the economy and then we're using too much in other parts of other economies, a developed economy. So ultimately, that's why. And we need protein, when there's too many people in the world, we're overusing, we're creating all sorts of offside impact because of that. So we need to have more global and circular food systems to actually support a growing population. Simple as that.” CoGB Employee, Senior Management |
| Participants consider the health and sustainability outcomes of our food system as a core responsibility of local governments | | “local governments have an important role in this space. And I guess it's a sort of an emerging field for local government to be working in, compared to where they have traditionally worked. But yeah, there's just so many parts of the food system that we know local governments have, I guess, touch points with.” CoGB Employee, Project Officer |

| *Domain* | **Characteristics of Individuals** | |
| --- | --- | --- |
| *Construct* | Self-efficacy | |
| *Description* | Individual belief in their own capabilities to adopt healthy and sustainable diet-related practices, as promoted within the food policy. | |
| *Sub-Themes* | | *Example of Illustrative quote(s)* |
| Participants describe personal motivations to adopt healthy and sustainable diet-related practices such as supporting local producers, contributing to the local economy, increasing local employment, the farmers’ market atmosphere and social connections, | | “I really like the idea of a sustainable, local commodity where we keep our money here, where we know what we're doing to our environment, where we know we're not impacting negatively on other systems like the Darling which we clearly are.” External Stakeholder (Local), Mid-Management  “And that's why I love farmers’ markets and the smells and the whole atmosphere of people with rough, dirty hands, because that goes back to my roots. And that's where I support them 100% in my work. I always make time for it because I understand the importance of it.” External Stakeholder (Local), Senior Management  “Because it relates to my background in food and agricultural production, but also understanding that we can do it differently and still make a good buck, but also do it in the right, ethically in the right way” CoGB Employee, Senior Management |
| Participants describe some barriers to adopting healthy and sustainable diet-related practices themselves, such as time constraints and accessibility of the farmers’ market. | | “You’ve got to think about agriculture's really a very broad industry. And while it's nice to think that agriculture, small-scale, local agriculture could be - you know, Bendigo could feed Bendigo, small-scale agriculture probably isn't feasible to feed everybody, because not everybody can afford to eat at the farmers’ market every week. That's why not everybody does. And not everybody has the time to - not everybody's able to finish half an hour early so that they can get to the farmers’ market to be able to do that.” CoGB Employee, Project Officer |
| Some participants describe their involvement in CoGB’s food sustainability agenda as facilitating their own sustainable practices | | “Three years ago, honestly. Look, I grew a veggie garden at home but I couldn't have cared less. Didn't know. Didn't care. Now, yeah. Now, I mean, my entire house, and professionally, like I volunteer at Foodshare now. I volunteer at soup kitchens. Out in our garden we've deliberately planted 500 tomato plants, 100 zucchinis, 100 capsicum, 100 cucumber. And I've lost count of how many lettuce, because we're not going to be able to grow all that in our gardens. So Gosh darn it, we're going to have to send every single student home with about a dozen seedlings. It's taken over.” CoGB Employee, Mid-Management |
| Other participants describe their historical adoption of healthy and sustainable diet-related practices, which possibly drew them to work in this area. | | “, I spent a lot of my time as a vegetarian growing up on a beef farm in northeast Victoria. It was part of the process from when Daisy stood in the paddock to when Daisy came to my plate, and I just found it very difficult to relate to all the smells and experiences along the way that we shouldn't eat Daisy, I couldn't do it. And interestingly enough, I've never - just within my own family, I've never really had a focus on meat. But my boys are like, “Where’s the meat? Where’s the meat? Where’s the meat?” I haven't done that at all. You know? I'm rather, “Where's the beans?” I have ownership of the work because of my upbringing. It's not something that I've had to buy into because I don’t understand it or I learned it through a book in university, if that makes sense. I grew up with it.” External Stakeholder (Local), Senior Management |

| *Domain* | **Characteristics of Individuals** | |
| --- | --- | --- |
| *Construct* | Personal Attributes | |
| *Description* | Participants’ personal traits such as leadership qualities (as opposed to organisational leadership), tolerance of ambiguity, intellectual ability, motivation, values, competence, capacity, and learning style | |
| *Sub-Themes* | | *Example of Illustrative quote(s)* |
| Participants described the leadership qualities amongst their colleagues and external partners as a critical driver to achieve food sustainability policy action | | “So our council is one of the councils that's declared a climate emergency…. The priorities in the environment strategy are in the health and wellbeing plan and in the council plan. So we've got a really close alignment. I've led that process… with my colleagues, because it's allowed me to work with colleagues across the whole council - and again, it's in the conversations… and a lot of it is about power dynamics, relationships, networks. That's how you… change people's mental models, and then allow them to change their policy practices and resource flows. So, that's really important. Culture is really important with all this stuff.” CoGB Employee, Senior Management |
| CoGB employs individuals in the health and food sustainability space with a breadth of experience in leadership (e.g. CEO and Director level experience), academia, legislation and advocacy | | “When I was working as a lawyer, I ended up working in Alice Springs with Aboriginal Land Council, which was a representative body of 90 elected members. And that was sort of at a time where a lot of the local organisations had been sort of undermined or disbanded through the Intervention. So while it was a land council and its work was around sort of land rights and native title, people were bringing issues like food security and access to healthy food and alcohol policy and education and everything to the mix.” CoGB Employee, Mid-Management |
| Individuals involved in CoGBs food sustainability agenda are bold, systems-thinkers. | | “Having been to Canada and spoken with xxx and obviously, when xxx came out from there to speak at the food hubs conference, just trying to bring those perspectives out that can share that broadly. It's basically about prevention. Rather than we just treat this as an outcome, the bottom of the cliff stuff, how can we actually work with generational shifts?” CoGB Employee, Senior Management    “But a bit like I said that all the council staff are health promotion workers, I also want the all the staff to be environment workers, because, again, the decisions they make - the procurement, the contracting, the way they approach where they choose to focus their effort and resources - can have an impact on the environment. And I think we're starting to sort of build that understanding.” CoGB Employee, Senior Management |
| Participants describe themselves as passionate about CoGBs food sustainability agenda. | | “I would say that my passion overrides – I’ve been called the most unprofessional professional many, many times, because I get excited, I get passionate about it. I know I start talking way too fast, and I get excited, and I can lose people sometimes, because I just get on a roll and I'm so keen about what it is that I'm doing, that I can lose people.” External Stakeholder (Local), Mid-Management |
| Several participants have practical agricultural experience (either working on or growing up on farms), which has motivated their current work in food sustainability. | | “So I'm originally off of a family farm. So potato farms actually. So way back I've got an agriculture degree, so a lot to do with food production. And also worked in the sector from a government perspective, and also worked in hospitality internationally. So done a whole heap of things around that and food, then being involved in the Gastronomy bid here, the UNESCO Gastronomy bid here in the city. But I'm primarily an agricultural scientist, my background is and my degree is there. But I've done all sorts of other things throughout the process and work all over the private sector, public sector, for the last 20 years.” CoGB Employee, Senior Management |

| *Domain* | **Inner Setting** | |
| --- | --- | --- |
| *Construct* | Goals and Feedback | |
| *Description* | The degree to which goals or intentions of the food policy are clearly communicated, acted upon, and fed back to stakeholders, and alignment of that feedback with the goals. | |
| *Sub-Themes* | | *Example of Illustrative quote(s)* |
| The goals and actions outlined in the Food Systems Strategy are clearly articulated and accurately represent community need however without adequate resourcing and government support, will remain “a great ambition”. | | “The accountability to get any of those goals, the really important objectives for public health, it sits with government. It has to even though - I've talked about that letter of political opportunity, you got the politics at the top and the community at the bottom. The community can entertain and be part of the agenda setting. But without the resourcing and the policy settings, you just can't get the outcome. So we've got a range of things happening at a regional level and a lovely action plan. You will save dozens and dozens of actions in the food system strategy and it will depend on, it's got desired outcomes and how would success look. A lot of that's dependent on investment and support. And it looks nice on a page and I'm not belittling it at all, it's a great ambition. But unless there is really decent resources devoted, it might get to where it needs to get and the proof of the pudding are basically, type 2 diabetes rates, all those things that are escalating. They keep escalating and keep escalating. And we've got lovely policies, et cetera, evolving, but not at the level at which it's going to get the resourcing in a government setting.” CoGB Employee, Senior Management |
| CoGB aims to establish a system that favours healthy, sustainable and equitable food then step aside and ensure system is continually evaluated and remains on track. | | “So I think the thing about local government is if you've got your system working really well, we can almost step out of the way. So we would probably always need some technical specialists to just continue to advise and coach and support people. So whether people with nutrition background, environment background, but people who understand systems thinking, those sorts of things, people who can make sure that we're getting the feedback, data, the evaluation to make sure we're continuing and we don't suddenly go off on a tangent in some strange direction. We would need those.  But a lot of people talk about you just - for a lot of things in the health space, you just throw more resources at it. I don't think we're ever going to get enough resources if we keep thinking that way. So it's really about once we've got these things in place, how we support them to stay until they basically become part of our culture, that we don't even think any differently. This is the way we do things around here in greater Bendigo. You have access to a healthy, sustainable equitable food system, and people understand that that's really important for their health and wellbeing, for their economic outcomes, and for the environment. I think that's what we're trying to do is drive cultural change.” CoGB Employee, Senior Management |
| CoGB plays a knowledge broker role, creating opportunities for residents and key stakeholders to provide feedback on the food policy and inform future policy action. | | “I see their role a lot as really connecting with the community at that local level to kind of connect things that are happening at a higher level, like might be policies and systems more at a state level or at a federal level and kind of bring it down and actually help to kind of operate – operationalise if that’s a word - some of those things. And I think that what we say a lot with the importance of local government is just having that real local relationship like they’ll - they know the food businesses in their area, they know the growers in their area, they know the local food economy and how that kind of works. So that they can kind of be, like I said, help to translate anything that might be happening at a higher level, if they can kind of then work on those projects and relationships more at that local level, obviously then feeds back up, back up the chain to that higher level as well.” External Stakeholder (State), Mid-Management |

| *Domain* | **Inner Setting** | |
| --- | --- | --- |
| *Construct* | Culture, politics and learning climate | |
| *Description* | Reference to the workplace culture within CoGB, specifically whether it values employees and nurtures curiosity, motivation and reflective thinking. Reference to internal tensions and 'tussles' as a result of these cultural norms and values. | |
| *Sub-Themes* | | *Example of Illustrative quote(s)* |
| CoGB fosters a culture of reciprocity and respect both internally and when working with external partners. | | “And I think it's more - rather than potentially saying we're operating in a collective impact model, it's more around saying we're operating under an agreed set of principles, that now I would say in Bendigo are just really embedded within the leadership. You know, xxx flicks me emails all the time and says, “What do you reckon? How about this? I want your insight,” and that sort of thing. And there's just that openness.” External Stakeholder (Local), Senior Management  “My managers are excellent in that they have said as long as you prepare a business case… with these ideas. They really work on the idea that if you can give us a good business case, we’ll support you. But also, make sure it is a good business case.” CoGB Employee, Project Officer |
| CoGB nurtures their workforce to apply evidence-based ways of working, for example systems-thinking | | “They've offered a lot of training on systems thinking. When they write up plans, they write it up and you would have seen them. They write it up. They present it as a system. So they use some really good tools, but they also have practice meetings, where people talk about - and they might have a - the last time we did - and they apply this beyond the food system. They applied it for our - we're working on the municipal health plan and they got someone in who's a systems expert. And she was brilliant. She was really fun. But doing that innovation, knowing where your risk level is, appetite is for innovation. And if you're going to do innovation, how are you going to evaluate that and make sure that you're not doing it - making things worse. So all of that they're very - they've got quite a sophisticated team in terms of systems thinking, so they're really cutting edge around that topic… they've invested a lot of time into understanding it and putting it into practice. It is the lens they look through now.” External Stakeholder (Local), Mid-Management |
| Tensions exist for some CoGB employees between fulfilling the traditional role of local governments and a more progressive approach, which may be less favourable for residents. | | “Yeah, there's always detractors who think it's just a sort of vanity project, or - yeah, it's just wasting time. But I think any project in a local government setting is going to have people saying just focus on roads, rates, and rubbish. And all of this international networking and stuff like that is - and creative industries stuff is a nice to have but isn't really core business. So yeah, I guess, more progressive councils try to explain to the community that that stuff is important. And it is. Now, local government does a lot more than it used to do in terms of trying to help with economic development, tourism, and things like that.” CoGB Employee, Project Officer |
| CoGB nurtures a workforce culture which challenges the status quo, for example embracing the arts to achieve systemic change and exercising agility regarding areas of focus (e.g. health vs sustainability) | | “I guess it's just about getting creative minds involved. So yeah, I think creative people sometimes can think differently about problems and how to solve them. And also, just getting involved in creativity brings a lot of wellbeing to people. So I guess in my utopian urban farming world, there's a lot of arts involved, as well as creative people coming up with how to design these urban farms. And there's, you know, creative people, documenting it and decorating it and communities involved in that. And that brings a lot of vibrancy and wellbeing to people as well. So yeah, I think supporting creative industries creates a happier community with more going on and want to get involved in.” CoGB Employee, Project Officer |

| *Domain* | **Inner Setting** | |
| --- | --- | --- |
| *Construct* | Structural characteristic & internal communications | |
| *Description* | The organisational structure, social architecture, age, maturity, and size of the local government. Nature and quality of internal social and formal communications networks. References to siloing and collaboration as a consequence of these structural or communication factors. | |
| *Sub-Themes* | | *Example of Illustrative quote(s)* |
| CoGB has adjusted their organisational structure to reduce siloing and reflect the evidential link between health and environment | | “And I can say we're moving towards that, with the Environment Team being brought right now into the Health and Wellbeing Directorate. And so we haven't seen how that will play out but I just think the nature of physically sitting in the same building, and that kind of thing. Having your managers all in the same meetings together, makes a really big difference in that type of thing.” CoGB Employee, Mid-Management |
| CoGB enjoys the benefits of a ‘small-town’ community and organisation as well as the resources of a larger town. | | “I think part of the benefit, because of the size of Bendigo, is that because we're not too big. So you actually kind of keep bumping into the same people all the time, so you kind of get to know each other. Whereas I think in a bigger place like Melbourne, that must be a bit more challenging. Most of us that work there, we also live there. So we're also community members as well. So I get the impression, and what I hear from others, is that we're in that nice size of a city. We're a small city but we've reached a sort of stage where we can have a lot of things that any bigger city has, but we have other benefits because we're not too big.” External Stakeholder (Local), Senior Management |

| *Domain* | **Inner Setting** | |
| --- | --- | --- |
| *Construct* | Internal policy, initiatives & incentives & Compatibility | |
| *Description* | The capacity of CoGB to execute the goals of the food policy, including available levers for change and opportunities to influence the food system to achieve health and sustainability outcomes. References to the legal remit of local government authorities (the core functions and legislative levers that exist) and how these are compatible/incompatible with the food policy. | |
| *Sub-Themes* | | *Example of Illustrative quote(s)* |
| Participants are optimistic in describing various opportunities available to local governments to address systemic food sustainability issues within their current remit. | | “Well, we have policies that cover public environments… healthy choices. We have a free implementation service that is freely available, so nobody can ring me up and say they don't know what to do with the policy. They can go to training, online training, they can Food Check it, there's a lot of services. There's a peer support coaching programme, so all of that's freely available, for childcare as well, everything, schools. So it's all there, it's up to people to use it. And my dream is that HEAS are run off their feet, because the more they get run off their feet, the more we can advocate for the need. So we've got that. We've got INFANT freely available as training for … professionals in Victoria. We've funded that right now. And dieticians, health promotion practitioners can do that. We've put the lever points in sport and rec funding. So new funding from sport and rec, which is millions and millions for new builds or retrofits, has to be meeting healthy choices. Let's see, what else do we do? Well, we've now just mandated the healthy choices policy and no sugary drinks in health services across the state. So that's just new as of August. And as everyone knows we're working up the local food procurement policy, is getting worked up for the state purchasing. We also have a policy in government that is binding, it's called the social procurement framework. And that requires government departments to prioritise social enterprises. In terms of whether it's disability or Aboriginal social enterprises, there's a preference to be supporting them. I actually feel we've got a lot of things in place. That and we've got the public health and wellbeing plan. So we've got a couple of bits of legislation.” External Stakeholder (State), Senior Management |
| CoGB has developed a solid foundation of relevant internal policy over many years, to which the food systems strategy builds upon | | “I think the main role the local government plays, and can play, is through strategy and policy development… I know the Victorian Public Health and Wellbeing Plan has climate change as one of the key action areas, and I'm assuming that will be reflected in local governments' health and wellbeing plans as well. The Healthy Together Bendigo work and the Greater Bendigo Food Security report that was developed identified food systems actions, both in the short and long term. Most of this work progressed throughout the Healthy Together Bendigo funded period, and a few actions from this report were then identified as still a priority and transferred into other Council plans. It was identified that that a dedicated food systems offer position is needed to progress key food systems work for the region.” CoGB Employee, Project Officer |
| Some participants describe addressing food sustainability as a non-traditional or ‘fringe’ issue for local governments’ to address | | “I say fringe because local government traditionally is quite a simple beast. But then, as communities have gotten more sophisticated and bigger, you're then able to sort of branch out to more interesting topics. Food and water and other things - food is probably, I suppose, what I’d consider a bit more - you know, from a traditional local government sense, it's a bit more fringe. It’s become a bit more mainstream now. But just by having that big organisation, we've had the capacity to, say, put on a food systems officer and develop food system strategies and things like that.” CoGB Employee, Mid-Management |
| Some healthy and sustainable practices are more clearly aligned or compatible with ‘core’ local government functions than others (e.g. waste management) | | “I think it’s a case of what local government can do, and where they feel they've got scope and power to act would be one explanation, I think, for that. So, a food systems strategy will almost always talk about local food and local food systems and promoting local food and produce sustainably. And increasingly talking about First Nations culture, and there is a link with economic development there. So, I can see why they’d prioritise that. In terms of why they wouldn't prioritise the recommendations on animal derived foods, and I guess, that would go along with the increasing plant-based foods. I'd imagine, without having researched it, that there might be lobbying factors and vested interests at play there that would, that they'd be aware of if they went out, and were basically telling people not – to really reduce their intake of red meats, let's say, or dairy.” CoGB Employee, Project Officer |

| *Domain* | **Inner Setting** | |
| --- | --- | --- |
| *Construct* | Access to resources, knowledge and information | |
| *Description* | The level of resources (e.g. money, training, education, physical space, and time) dedicated to food policy action. Accessibility to digestible and credible information about community needs, evidence-based policy action and best-practice implementation and evaluation. | |
| *Sub-Themes* | | *Example of Illustrative quote(s)* |
| CoGB is strategic in accessing knowledge and information, by actively seeking opportunities to learn new, evidence-based ways of working to achieve the best outcomes. | | “Council… truly understand the community and the strengths of the community so that they don't - they're not the authority and they're not always the experts. I think it performs the function performs best when they look for partnerships… by supporting it in the right way, not necessarily pulling or tugging or trying to create what they think. Taking advantage of the strengths of that community group, of that organisation of those people, to be their best, to deliver the best outcome for the community. And that would maximise their dollar investment too.” External Stakeholder (Local), Senior Management |
| CoGB’s historic investment in community engagement, academic partnerships and data collection enabled access to information about community need and prioritisation within the food sustainability agenda. | | “. And yeah, we also do a lot of community consultation. We’re sort of mandated to do community consultation in regards to all of our work, so we do get a lot of feedback from the community and they do help in giving us a better picture of what we might prioritise that will address their needs.” CoGB Employee, Project Officer  “Obviously, the environmental sustainability work, the city of gastronomy stuff had been happening behind the scenes. We'd had the first Active Living Census, and we’d completed the work through Healthy Food Connect. So we had the food security scan. Yeah, so I guess a whole range of things had been chipping away along the way that were brought together.” CoGB Employee, Mid-Management |
| Participants consider CoGB better resourced than other local government areas, yet participants in higher levels of management describe a shortfall in resources for the food system strategy | | “And we don't get any real funding from government for… the systems, food system strategy. The money for the resourcing, the sort of backbone… is really funded by council. So in one level, that gives us authorised environment to do what we think is the right thing. And we'd looked - I look at other literature, I look at overseas… VicHealth is going down this way of thinking as well. So we've had a lot of conversations with Sandro and others about our work. And they're doing some different sort of models of systems thinking as well. So I think it has to be the way, because you can't throw bits of money at things and then expect everything to change; you have to change the way we sort of almost live our life… Even if everyone in council was working on food, it wouldn't be enough. So we have to engage the community in this way of thinking.” CoGB Employee, Senior Management |
| CoGB employs well-connected and highly skilled individuals who can access credible evidence. | | “Bendigo is a real cauldron of passionate people who they've had their experience in Melbourne, I guess, and then they've come to Bendigo for, I don't know, the beautifulness of it all. Really skilled, passionate people... Just people got it and understood how it all fit and it all connected together. And I think that because of - yeah, it's just the coming together of all the minds that’s enabled it to happen.” External Stakeholder (Local), Senior Management |

| *Domain* | **Inner Setting** | |
| --- | --- | --- |
| *Construct* | Effective leadership and internal champions for change | |
| *Description* | Reference to the influence of individual people or ‘champions’ (CoGB employees) to prompt food policy action. The degree to which leaders and managers are perceived to be committed, competent and accountable for the food policy and effectively engage and inspire their colleagues and external stakeholders. | |
| *Sub-Themes* | | *Example of Illustrative quote(s)* |
| CoGB strive to lead by example, and in doing so test the waters for others | | “We've led the way with some of our own policies. So a lot of our work, we start by looking at our own practice first. So whether it's a safe, healthy food and catering policy. It’s a bit rich to go out and ask someone else to do something if you're not doing it yourself. And along the way, we usually learn some stuff about what works and what doesn't work before we start going out. ” External Stakeholder (Local), Senior Management |
| The workplace culture at CoGB supports bold leadership and are considered ‘early adopters’ | | “Absolutely, good quality workforce that’s surrounded by opportunity. The leadership in the town is willing to try things and to be innovative.” External Stakeholder (Local), Senior Management  “I think number one is a level of governance, where boldness is valued. And risk aversion is certainly not a consideration. I think that would be huge for local governments to make progress. And I guess that is the challenge that we face is that local governments being very bureaucratic in nature, quite often stifling innovation by all of this risk aversion that seems to come naturally into our environment. So certainly boldness.” CoGB Employee, Mid-Management |
| CoGB has a history of employing leaders who are equipped with food system knowledge, passion to improve the system and a sense of community accountability | | “Definitely having people in those leadership positions who understand food systems and the environmental impacts of food. Also having a community who push for action in that area.” CoGB Employee, Project Officer  “They've always had players, strong players within local council, employed by local council, not the councillors, they're different, who have had a really big focus on doing well for their community.” External Stakeholder (Local), Mid-Management |
| Effective leadership requires those in higher levels of authority to set the agenda and resource the work, while simultaneously inspiring champions throughout the organisation to execute the agenda. | | “Probably not, we probably get that push more from our Director of Health and Wellbeing. She's a really strong advocate, especially in the food space. She's a dietitian as a background, so she's been a really strong support for us to do more. And our CEO probably provides that supportive environment where he will back his staffing when we do try to put forward kind of new ideas that we have been doing.” CoGB Employee, Mid-Management |
| Participants described a number of ‘champions’ working within CoGB with key skill-sets | | “I guess yeah, there hasn't been anything that I've gone to her (xxx) with that she hasn't been able to come back with the right person or the right advice. Yeah, so look, very knowledgeable, very passionate, a great networker. Look, they’re definitely qualities that she's got. I guess, humbles not the right word. She doesn't profess to know much about it at all, which is actually really nice, but you don't go in there feeling like an idiot. You go in there feeling like you're on par with them. And that's, I guess it's a really – it's good that you don't intimidated, you don't feel pressured. I've never felt pressured to push anything she's thrown my way. But she's also relentless. And any little thing that she thinks that could be right in my alley, I get the email. Or anything that she thinks that I could do that would help her out, well, look, I jump. I jump at the opportunity to help her because as I said, most of what my curriculum is now is based on the work that I'm doing with her. So, I need to be thanking her. But yeah, look, she's great at relationships. It started off as a very, very professional relationship, like I’d call her a friend now. It's been really nice like that, that you can just bounce ideas off. Yes, so, like I said, that relationship building, networking, really, really strong.” External Stakeholder (Local), Mid-Management |

| *Domain* | **Inner Setting** | |
| --- | --- | --- |
| *Construct* | Readiness for implementation and tensions for change | |
| *Description* | Indicators that the CoGB is committed to taking action on food sustainability. The degree to which participants believe the pre-policy situation to be intolerable or needing change. CoGB's willingness and capacity to develop, implement and evaluate the food sustainability policy - including mention of aspirational values and the appetite for innovative approaches. | |
| *Sub-Themes* | | *Example of Illustrative quote(s)* |
| CoGB is walking a tightrope between being ‘liked’ by the community and implementing evidence-based, bold and “controversial” policy action. | | “That's not to say that meat and three veg is probably a great cultural thing to uphold. But in terms of if that's how people have been normalised it's, I guess in short, we're not trying to take away people's Christmas turkey or things like that. So how to try and balance some of those tricky issues where it's – well, we need to do something, so business as usual is not going to work, but how do we make it work for you?” CoGB Employee, Mid-Management |
| Social injustice and threats to planetary health are described as tensions which have prompted and drive CoGB’s food policy work | | “Their core business still is around finding food to be able to distribute. They’re also looking at how they can be part of the solution for people to no longer require more emergency food. How do we help people out of that cycle? So that's part of their focus now too.” CoGB Employee, Project Officer  “We've been trying to create a movement for health and wellbeing, and it's not the site, it's not real, you know, it's not that sort of grassroots, the people are demanding it. So I think that's where the shift will come from. And obviously, the existential risks to the entire planet.” CoGB Employee, Mid-Management |
| Participants describe the delicate tension between their commitment to value and support key stakeholders, including local industrialised primary producers, and deliver bold food sustainability policy actions | | “And I think there's maybe a little bit of - potentially a sort of conflict between some of the bigger and perhaps less sustainable agricultural industry that exists in our region, which we also kind of used strategically in our UNESCO application to say, you know, agriculture, and associated industries are a huge employer. It's a huge part of our region. But at the same time, we're wanting to talk about smaller scale, artisanals, more sustainable practices. So sometimes that can be a little bit - it’s a bit jarring, trying to support both.” CoGB Employee, Project Officer |
| CoBG works with the community to address tensions and pushbacks where they believe the outcome will ultimately be beneficial | | “But we what we've gradually done is by building partnerships, and understanding and credibility, we're gradually taking them on a journey towards food... But you've got to start where people are at, and you also have to realise why they do certain things. So, sports clubs, for instance, why do they have all these deep fryers? Because they sell a lot of chips and that's their fundraising. And so, just trying to get - to sort of build that understanding that actually, that doesn't really align with the rest of your ethic of your club. You're trying to get social connection and physical activity and health and all of that, and then you feed them deep fried chips. So there's a whole range of - you have to sort of take people on this sort of thinking journey, really.” CoGB Employee, Senior Management |
| The levers available to CoGB and the election cycles challenge their commitment to meet community needs and achieve health, sustainability and equity outcomes | | “The Food Act is certainly working against the type of healthy eating messages that we're trying to get out there into our community by making the perhaps the unhealthy choice, the easiest choice.” CoGB Employee, Mid-Management  "We say we're about local community members as part of the decision making process but realistically, policy procedure legislation means that they always a couple of steps away. So I think community engagement right at the core of local government, in their decision making, would lead to a utopian state of community ownership." CoGB Employee, Mid-Management |
| CoGB and other progressive local governments have an appetite to apply the sustainability lens to their already familiar health lens | | “But a lot of people are asking questions around, “If I want to incorporate things around sustainability, how do I do that?” So, there's definitely interest in it. I think there's still - I think local governments are still trying to figure out I guess what they can do or how they can do it. They seem to know a bit more around – it’s a bit easier for them to understand what they need to do on a health level, but I think the sustainability side of things, I think, seems like they're still trying to understand how they could have a role” External Stakeholder (State), Mid-Management |

1. Nolan MB, Warner DO. Perioperative tobacco use treatments: putting them into practice. BMJ. 2017 Sep 6; 358: j3340. doi: 10.1136/bmj.j3340. PMID: 28877905. [↑](#footnote-ref-1)
